# Evidence for a post-invasion role of the *Chlamydia trachomatis* type III secreted effector TmeA in redirection of host plasma membrane-derived material

**DOI:** 10.1128/mbio.01993-25

**Published:** 2025-09-25

**Authors:** Phuhai Nguyen, Caroline Hawk, Katerina Wolf, Kenneth A. Fields

**Affiliations:** 1 Department of Microbiology, Immunology and Molecular Genetics, University of Kentucky College of Medicine 214561, Lexington, Kentucky, USA; University of Nebraska Medical Center, Omaha, Nebraska, USA

**Keywords:** TmeA, lipid, vesicle trafficking, obligate intracellular

## Abstract

*Chlamydia* spp. infect mucosal epithelia and develop within a membrane-bound vacuole termed an inclusion. Parasitism of resources from host organelles and exploitation of the actin-based cytoskeleton represent central paradigms in the establishment and maintenance of the intracellular niche created by *Chlamydia* spp. Manipulation of host cell biology is mediated in large part by deployment of type III secreted effector proteins. Translocated membrane-associated effector A (TmeA) is a type III secreted effector first deployed during invasion, where it is necessary for efficient entry into host cells. We provide evidence herein that TmeA also functions later during infection. *De novo*-synthesized TmeA is secreted from mature inclusions, and deletion of *tmeA* correlates with decreased inclusion size and progeny production. In addition, functional evidence supports a role of TmeA in intracellular development. We leveraged the lipophilic tracer DiI to reveal TmeA-dependent trafficking of plasma membrane-derived material to the chlamydial inclusion. The pathway was distinct from that described for sphingomyelin delivery to inclusions. Trafficking of DiI to the inclusion instead required actin polymerization and the TmeA Neural Wiskott-Aldrich syndrome protein (N-WASP) interaction domain. Ectopically expressed TmeA co-localized with internalized DiI in vesicle-like structures that also depended on the N-WASP binding domain. Clathrin and dynamin were also required but likely contribute independently of direct TmeA-mediated activation. Overall, our data provide evidence that *Chlamydia* repurposes TmeA to accomplish development-specific tasks and parasitizes the host surface to support intracellular growth.

**IMPORTANCE:**

*Chlamydia trachomatis* is a human pathogen and a prevalent agent of sexually transmitted diseases. The ability to survive and propagate within a protected intracellular niche leads directly to pathology indicative of *Chlamydia*-mediated disease. The reduced chlamydial genome leads to comparatively limited biosynthetic capacity, thereby necessitating parasitism of metabolites and other resources from the infected host cell. *Chlamydia* relies heavily on type III secreted effectors to interface with and co-opt host pathways to acquire resources. We demonstrate herein that the plasma membranes of infected cells represent a potential reservoir of resources required for optimal intracellular growth. Chlamydiae employ at least one type III secreted effector protein, translocated membrane-associated effector A (TmeA), to redirect material to the vacuole by manipulating Arp2/3-dependent actin polymerization. This pathway represents a distinct mechanism by which *Chlamydia* acquires resources and provides evidence for TmeA function during intracellular development.

## INTRODUCTION

Bacterial pathogens that replicate intracellularly have evolved elaborate strategies to invade and extract resources from infected host cells while avoiding cell-intrinsic protective mechanisms. Some, like *Legionella pneumophila*, secrete large subsets (>300) of anti-host effector proteins to accomplish this complex feat (1). Other pathogenic bacteria, including obligate intracellular *Chlamydia* spp., often rely on fewer effectors. *Chlamydia trachomatis* is a strict human pathogen divided into serological variants responsible for ocular infections leading to endemic blinding trachoma (serovars A–C) and localized (serovars D–K) or invasive (lymphogranuloma venereum, serovars L1–L3) sexually transmitted genital infections (2). Regardless of tissue tropism, epithelial cells represent an important replicative niche for chlamydiae. *C. trachomatis* actively invades these non-professional phagocytes and develops within a parasitophorous vacuole termed an inclusion. Developing inclusions excludes interactions with potentially restrictive host pathways such as the endo-lysosomal system (3). This intricate infection biology must be accomplished relying on a reductionist genome (4).

The infected host cell represents an important source of nutrients. As an obligate intracellular pathogen that is auxotrophic for many metabolites and building blocks (4), host cell parasitism represents a major theme for *Chlamydia*. Vacuoles occupied by other obligate intracellular pathogens, such as *Ehrlichia* or *Coxiella*, fuse with elements of the endolysosomal or autophagic systems to acquire resources (5). *Chlamydia* inclusions, however, restrict fusion with these compartments and must derive resources such as lipid-containing material elsewhere (3). Importantly, host-derived lipids are required to supply the expanding inclusion membrane (IM) and represent a significant component of the chlamydial envelope (6). For example, the chlamydial envelope contains lipids such as phosphatidylcholine, sphingomyelin, and cholesterol that are typically found in eukaryotic cells and are not *de novo* synthesized by chlamydiae (7–9). Selective interaction with host organelles represents an important mechanism for delivery of these host-derived lipids to intra-inclusion chlamydiae (6). Described pathways whereby lipids are delivered to the inclusion include interception of Golgi-derived exocytic vesicles (10), fusion with multivesicular bodies (11), engulfment of lipid droplets (12), or transfer of lipids from the endoplasmic reticulum (ER) through discrete contact sites or recruitment of sphingomyelin synthases to close proximity with the inclusion (13, 14). Fluorescently tagged lipid derivatives or fluorescent probes have been used effectively to define these pathways. For example, visualization of sphingomyelin transfer from the Golgi (10) or ER was accomplished using N-{6-([7-nitro-2-1,3-benzoxadiazol-4-yl]amino)hexanoyl}-D-erythro labeled ceramide, whereas filipin staining revealed the Golgi (15) and multivesicular bodies (16) as sources of cholesterol. In both cases, lipid-containing vesicles fuse with the inclusion membrane, where they are extracted by the bacteria and incorporated into the envelopes of chlamydiae (6).

Expression of a virulence-associated type III secretion system represents one mechanism used by *Chlamydia* to accomplish manipulation of host cell biology (4, 17). *Chlamydia* spp. manifest a biphasic development cycle alternating between metabolically dormant infectious forms termed elementary bodies (EBs) and metabolically active but non-infectious forms called reticulate bodies (RBs). Gene expression follows a temporal pattern corresponding to development, where subsets of the 894 genes in the genome become transcriptionally active during the early cycle (ca. 1–4 h), during the start of active replication during mid-cycle (ca. 12 h), or during late-cycle (ca. 15–18 h) when RBs begin to asynchronously differentiate into EBs for subsequent rounds of infection (18). An EB-localized T3S apparatus mediates secretion of effectors required for invasion and early-cycle development (19), whereas *de novo* expression begins during mid-cycle when RBs first begin to divide (20, 21) and contributes to subsequent requirements. A subset of so-called invasion-related effectors is prepackaged during EB formation and mediates entry-related events punctuated by manipulation of the actin-based cytoskeleton (22). A comparatively large class of integral membrane proteins that intercalate into the surrounding inclusion membrane predominates effectors that are expressed and secreted by intracellular chlamydiae. These inclusion membrane proteins (Incs) support multiple aspects of intracellular development due to their positioning at the pathogen-host interface (3).

The translocated membrane-associated effector A (TmeA) has been characterized as one of the invasion-related T3S effectors that is pre-packaged in EBs to support subsequent rounds of infection (23). This pool of TmeA is secreted during entry (23), and null strains lacking *tmeA* exhibit reduced invasion efficiency (24, 25). Once secreted, TmeA associates with host membranes and partitions with membrane proteins in biochemical fractionation studies (26). TmeA functions synergistically with an additional invasion-related effector, the translocated actin-recruiting phosphoprotein (Tarp), to promote actin polymerization. Loss of either effector results in decreased invasion rates, manifesting as a ca. 50% reduction in internalized EBs at 30 min (25). Reduced invasion rates are modest and likely reflect the noted existence of redundant entry mechanisms (3, 27) capable of compensating for loss of a single pathway. Tarp binds actin directly to promote polymerization (28) but can also recruit the actin-related protein complex (Arp2/3) to promote assembly (29, 30). TmeA contributes distinctly via interaction with host Neural Wiskott-Aldrich syndrome protein (N-WASP) to promote actin polymerization (25, 31). TmeA and Tarp can also promote Dynamin 2 (Dyn2)-dependent fission of nascent vesicles (32) to facilitate entry. The mechanism by which TmeA stimulates Dyn2 oligomerization is unknown but requires initiation by Tarp (32). Interestingly, ectopic expression studies using fluorescent or enzymatic reporters suggest that TmeA can also be secreted by intracellular chlamydiae within mature inclusions (33, 34), thereby raising the possibility of a post-invasion function for TmeA. We previously demonstrated that loss of TmeA manifested a modest decrease in intracellular growth, but this was a polar mutant that also lacked TmeB (24).

We provide evidence herein that TmeA also functions during intracellular development. *De novo* expressed TmeA is secreted by mature inclusions, and loss of *tmeA* reduces intracellular growth. The use of a lipophilic dye demonstrated that *Chlamydia* redirects lipid-containing material from the plasma membrane to the inclusion, independent of Golgi-derived lipids. TmeA contributed significantly to tracer trafficking, but not Golgi-derived sphingomyelin, via activation of N-WASP. This pathway is important for intracellular chlamydial growth since interference with host factors or loss of TmeA negatively impacted fitness. In aggregate, we provide evidence that select material endocytosed from the plasma membrane is directed to the chlamydial inclusion and that TmeA-mediated activation of N-WASP and the Arp2/3 complex contributes to this process.

## RESULTS

### Characterization of late-cycle TmeA

**Fig 1:**
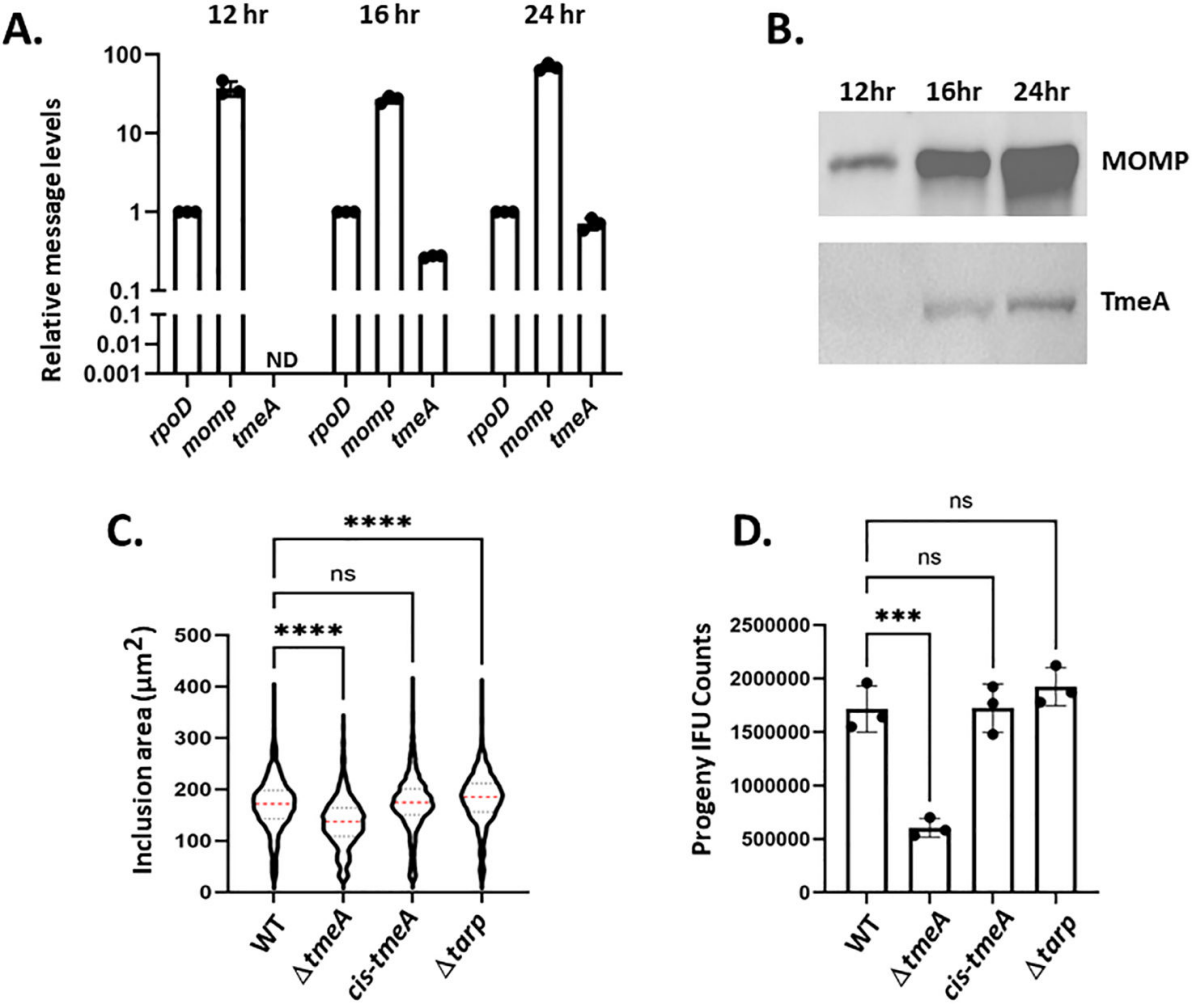
Evidence for TmeA function during late-cycle development. HeLa cells were infected at a multiplicity of infection (MOI) of 1 with WT serovar L2, and whole-culture RNA or protein was harvested at 12, 16, or 24 h post-infection. (**A**) TmeA-specific mRNA was detected via qRT-PCR and normalized to *rpoD* levels in triplicate replicates. MOMP-specific mRNA was similarly quantitated as a quality control. ND = none detected. (**B**) TmeA was visualized by immunoblot, where MOMP-specific signals were examined as positive controls. (**C**) HeLa cells were equally infected at an MOI of 0.5 with WT, Δ*tmeA*, cis-*tmeA*, or Δ*tarp Chlamydia* and cultured for 24 h. Cultures were methanol fixed and stained using Hsp60-specific and Alexa-conjugated primary and secondary antibodies, respectively. Inclusion images were acquired, and inclusion areas (µm^2^) were quantitated using automated CX5 software. Violin plots depict data plots (*n* = 1,000) from representative replicates. Mean values (red line) and SD (dotted lines) are shown, and one-way ANOVA with multiple comparisons was used to assess statistical significance (ns = not significant; ****, *P* < 0.0001). (**D**) Progeny EBs from triplicate HeLa cultures equally infected with WT, Δ*tmeA*, cis-*tmeA*, or Δ*tarp Chlamydia* were enumerated after 24 h cultivation of passaged material. Progeny count data are represented as means of primary culture replicates (closed circles) with corresponding SDs. Statistical significance was determined by one-way ANOVA with multiple comparisons (ns = not significant; ***, *P* < 0.001).

Secretion of ectopically expressed TmeA-reporter fusions in *C. trachomatis* has implicated a role for TmeA beyond chlamydial invasion (23, 33, 34). We examined TmeA abundance, secretion, and fitness of a Δ*tmeA* strain to address the possibility of late-cycle TmeA function more directly. Whole-culture RNA or protein was harvested from cultures infected with wild type (WT) serovar L2 at 12 h, 16 h, and 24 h post-infection and analyzed for *tmeA* expression (Fig. 1A and B). Transcripts were detected by quantitative reverse transcription polymerase chain reaction (qRT-PCR), and values were normalized to constitutively expressed *rpoD*. Consistent with previous data (21, 23), TmeA mRNA (Fig. 1A) was detected at times corresponding to the late-cycle temporal class of gene expression (20, 21) but below detection in mid-cycle (12 h) cultures. The message for the mid-cycle gene encoding the abundant major outer membrane protein (MOMP) served as a quality control and was detected at all time points as expected. TmeA was also detected in corresponding immunoblots (Fig. 1B) beginning at 16 h. Finally, we tested the fitness phenotype of our Δ*tmeA* strain by quantitating inclusion area (Fig. 1C) and enumerating infectious progeny production (Fig. 1D) from 24 h cultures in comparison to WT and a cis-complemented control. We confirmed equal inclusion numbers in the beginning primary cultures to mitigate potential invasion effects. We also included Δ*tarp* as an additional control since Δ*tmeA* and Δ*tarp* exhibit similar invasion rates. Deletion of *tmeA* led to significantly smaller inclusions at 24 h, whereas Δ*tarp* inclusions were slightly larger than those of WT and cis-*tmeA* strains. Likewise, Δ*tmeA* exhibited a significant decrease in recoverable progeny compared to WT and complemented control. This decrease was also manifested when progeny were enumerated from 48 h cultures (Fig. S1A). Invasion efficiency likely did not contribute to this decrease during passaging since Δ*tarp* progeny was similar to WT. In addition, particle-normalized infections resulted in an equal ability of Δ*tmeA* and WT to form inclusions (Fig. S1B), indicating that decreased invasion does not translate to the observed progeny reduction.

Despite evidence that TmeA is secreted by mature inclusions, apparent low abundance has confounded immunolocalization-based detection of endogenous TmeA outside of the inclusion.

**Fig 2:**
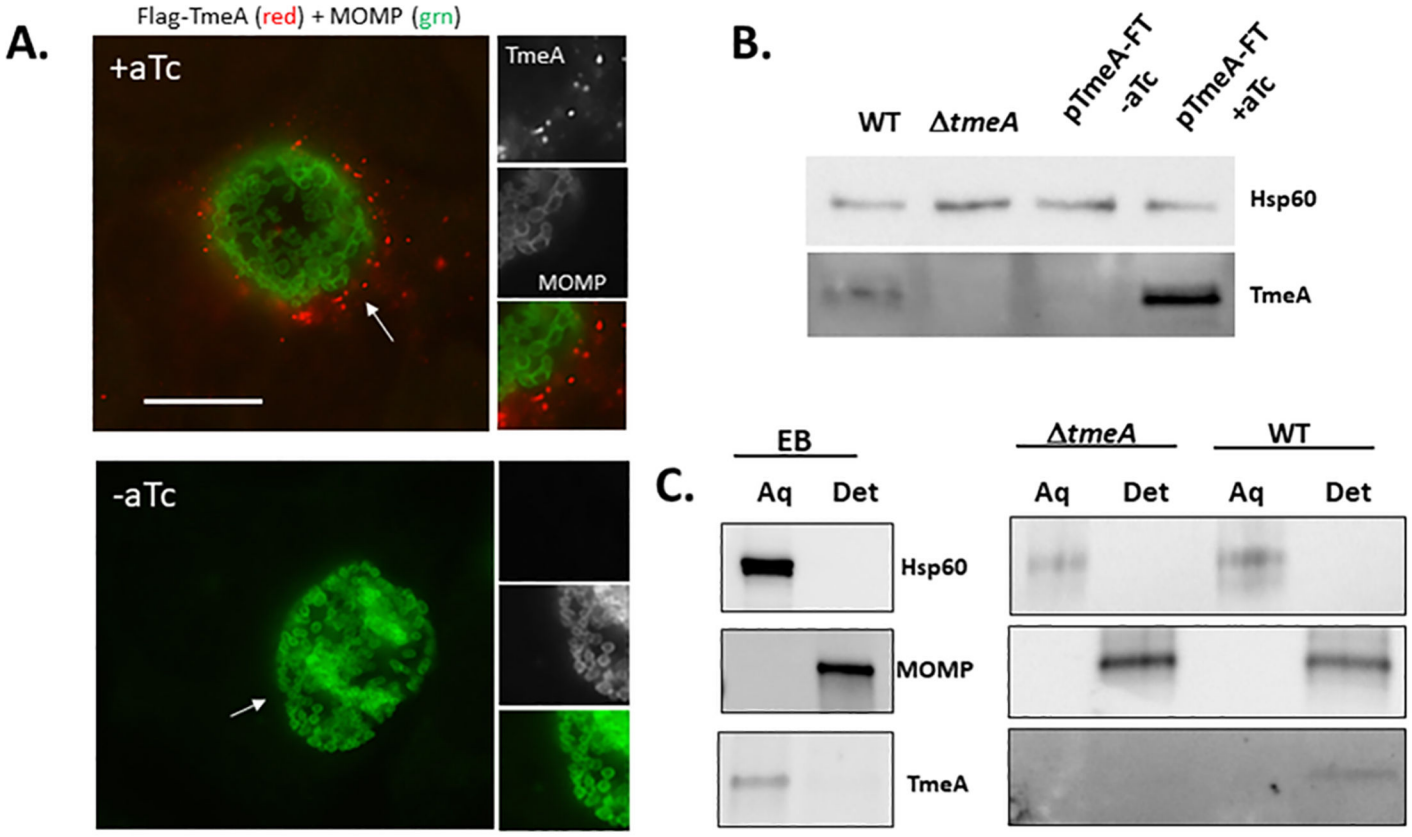
Secretion of TmeA by mature inclusions. HeLa cells were infected with Δ*tmeA* expressing pBOMB-TmeA-FT. Media were supplemented with inducer (+aTc) or mock treated (−aTc) at 12 h post-infection and processed at 24 h post-infection. (**A**) Cultures were fixed with paraformaldehyde followed by ice-cold methanol. TmeA and MOMP were detected using Flag (red) or MOMP (grn)-specific antibodies, respectively. Arrows indicate the area of inset and bar = 10 µm. (**B**) Immunoblot of whole-culture material probed with TmeA-specific antibodies or Hsp60 antibodies as a loading control. (**C**) Immunoblot analysis of endogenous TmeA was performed on Triton X114 aqueous (Aq) or detergent (Det) fractions of pure WT EBs or infected HeLa cultures harvested at 24 h post-infection. Antigen-specific antibodies were used to detect TmeA and Hsp60, and MOMP was visualized as a control for soluble and membrane-associated proteins, respectively. Fractionation of Δ*tmeA*-infected cells was performed as an antibody specificity control.

To overcome this barrier, Flag-tagged TmeA was ectopically expressed in Δ*tmeA* to enhance TmeA abundance. Expression was controlled by the *tet*-inducible promoter, and monoclonal Flag-specific antibodies were used to reduce background signal via indirect immunofluorescence. TmeA expression was induced with anhydrotetracycline (aTc) at 12 h post-infection and visualized 12 h later. TmeA-Flag signal was detected in punctate loci arranged at the inclusion periphery in ca. 20% of infected cells. (Fig. 2A). Immunoblot analysis of whole-culture material with TmeA-specific antibodies confirmed robust levels of TmeA in the presence, but not absence, of aTc (Fig. 2B). At later times (30 h), punctate TmeA-Flag staining was routinely detected within the host cytosol, whereas the cytoplasmic control protein NrdB-Flag co-localized with chlamydiae-specific MOMP signal (Fig. S2). These data confirm secretion of *de novo* synthesized TmeA during later times of development and reveal a potential vesicular pattern. Triton X114 partitioning was used to test the secretion of endogenous TmeA since we have established that secreted TmeA associates with host membranes and partitions to the detergent phase (26). Purified WT EBs or 24 h infected cultures were extracted with Triton X114 and partitioning analyzed by immunoblot (Fig. 2C). Extraction from Δ*tmeA*-infected cells served as a negative control. As expected, EB-localized TmeA partitioned to the aqueous phase. In contrast, TmeA was detected in the detergent phase in infected culture material, a localization indicating secretion. Any EB-derived TmeA in the aqueous phase was below detection at this time point. Hsp60 and MOMP were visualized as controls for soluble and membrane-associated proteins, respectively. Overall, these data are consistent with TmeA exerting a functional role during the intracellular development of growing inclusions.

### TmeA function during intracellular development

**Fig 3:**
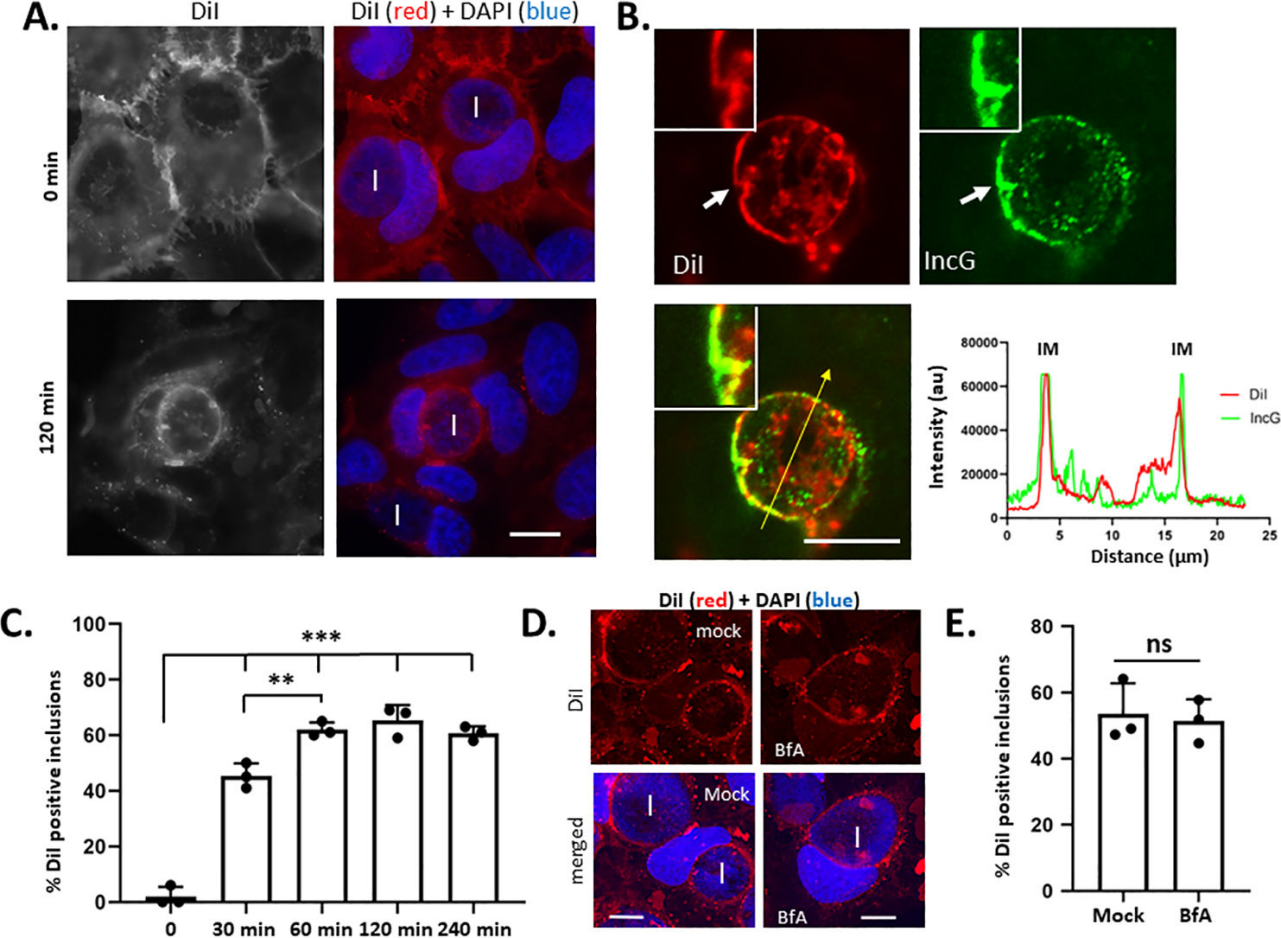
Surface DiI is trafficked to WT inclusions. HeLa cells were infected with *C. trachomatis* L2 and pulsed with DiI for 5 min at 24 h post-infection. (**A**) Epi-fluorescence images of DiI-pulsed cultures at the time of dye removal (*T* = 0) or after incubation for 120 min. DiI is visualized as gray/red, while inclusions (I) and nuclei are shown in blue in merged images. Bar = 10 µm. (**B**) Confocal images of infected HeLa cells paraformaldehyde-fixed 120 min after DiI pulse. Saponin-permeabilized cells were counterstained with IncG-specific antibodies. White arrows indicate the inset region, and the yellow arrow indicates the plane for quantification of intensity profiles plotted as arbitrary units (au). The position of the IM is indicated on intensity profiles. Bar = 10 µm. (**C**) Inclusions (*n* = 100) were scored for co-localization with DiI over times ranging from 0 to 240 min post DiI pulse. Data are presented as means of triplicate replicates (closed circles) with SD, and one-way ANOVA with multiple comparisons was used to assess significance (**, *P* < 0.001; ***, *P* < 0.0001). (**D**) DiI localization 60 min after DiI pulse of 24 h WT-infected cultures that were untreated (mock) or treated with 3 µg/mL Brefeldin A (BfA) added beginning at 4 h post-infection. DiI (red) and DAPI (blue) images are shown, and inclusions (I) are indicated in the merged images. Bar = 10 µm. (**E**) DiI co-localization with inclusions (*n* = 100) was quantitated, and average values for triplicate samples (closed circles) are shown with error bars corresponding to SD. Statistical significance was tested using Student’s *T* test (ns = not significant).

Decreased progeny manifested by Δ*tmeA* and the punctate staining pattern of secreted TmeA-Flag suggest a role in the trafficking of resources to the chlamydial inclusion. Studies addressing mechanisms governing the trafficking of lipids from host compartments to the chlamydial inclusion have leveraged specific fluorescent lipid analogs (6). Lipophilic dialkylcarbocyanine dyes have been used to trace lipid movement more broadly from the host plasma membrane to intracellular compartments during intracellular infections (35). Based on these observations, we employed the fluorescent, lipophilic molecule 1,1′-dioctadecyl-3,3,3',3′-tetramethylindocarbocyanine perchlorate (DiI) to test whether a generalized mechanism exists for lipid-containing material to be mobilized to the chlamydial inclusion. HeLa cells were first infected with *Ctr* L2 for 24 h and then pulsed at 4°C with DiI to selectively label the host plasma membrane. Cultures were shifted to 37°C to allow endocytosis, fixed at the time of shift or 120 min post shift, and counter-stained with DAPI for epifluorescence analysis (Fig. 3A). DiI signal was concentrated in HeLa plasma membranes when cultures were fixed immediately after temperature shift, but co-localized at later times with most, but not all, inclusions in rim-like patterns indicative of accumulation at or within the IM. A similar result was observed when non-transformed human endocervical epithelial cells (A2EN) were used as hosts (Fig. S3A). Experiments were repeated with counterstaining of inclusions with IncG-specific antibodies and visualization via confocal microscopy to more precisely discern DiI signal localization. DiI signal overlapped with IncG in the inclusion membrane, indicating that a pool of lipid incorporates into the IM (Fig. 3B). In subsequent experiments, this rim-like DiI pattern was scored as a DiI-positive inclusion. In time-course experiments, rim-like DiI staining appeared as early as 30 min after temperature shift, peaked at 60 min, and did not change significantly with longer incubation times (Fig. 3C). Although DiI was sporadically detected within the inclusion lumen, this signal did not appear to co-localize with IncG-labeled bacteria (Fig. 3B). Cultures were incubated with DiI from 4 to 24 h post-infection to increase DiI abundance. This treatment also resulted in co-localization of DiI with the IM but not individual chlamydiae (Fig. S3B). This was surprising given that specific lipids and cholesterol trafficked from the Golgi and/or endoplasmic reticulum robustly incorporate into chlamydial membranes. We used Brefeldin A (BfA) treatment to block the well-described Golgi-derived transfer of sphingomyelin to address this disparity. DiI co-localization with the chlamydial inclusion was not inhibited when BfA was added 1 h prior to and during DiI treatment (Fig. 3D) and quantitation of DiI trafficking to inclusions of mock or BfA-treated cells revealed no difference (Fig. 3E). In contrast, similar treatment with BfA interfered with inclusion labeling (Fig. S4A and B) when host cells were pulsed with N-WASP binding domain (NBD)-ceramide as reported (10).

**Fig 4:**
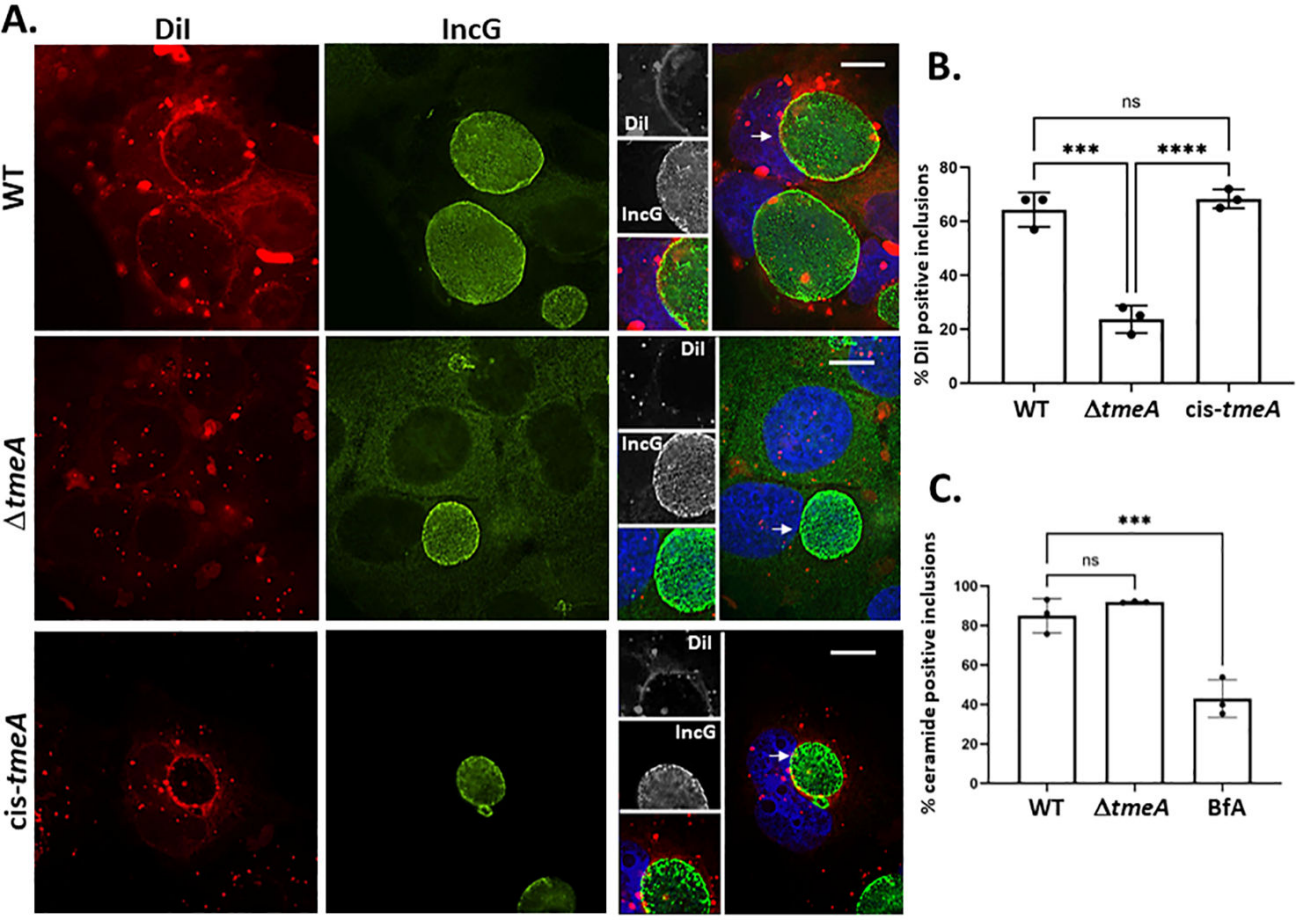
TmeA is required for delivery of surface-localized DiI to the chlamydial inclusion. (**A**) Representative fluorescence images of HeLa cells infected for 24 h with WT, Δ*tmeA*, or cis-*tmeA* and paraformaldehyde fixed 120 min after DiI pulse. Samples were saponin permeabilized and stained with IncG-specific antibodies and with DAPI to visualize nuclei. DiI (red) or IncG (green) signals are shown, and DAPI (blue) is shown in merged images. Insets from merged channels are included with gray-scale images. Bars = 10 µm. (**B**) Inclusions (*n* = 100) were scored for the presence or absence of IM-localized DiI. Data are presented as means of triplicate samples (closed circles) with SD. One-way ANOVA with multiple comparisons was used to assess significance (***, *P* < 0.001; ****, *P* < 0.0001). (**C**) Quantitation of NBD-ceramide co-localization with inclusions (*n* = 100) after 120 min in HeLa triplicate cultures (closed circles) infected for 24 h with *C. trachomatis* WT or Δ*tmeA*. Labeling of WT infections in the presence of 3 µg/mL BfA served as a positive control. Data are presented as means of triplicate samples (closed circles) with SD. One-way ANOVA with multiple comparisons was used to assess significance (ns = not significant; ***, *P* < 0.0001).

We directly tested the potential role of TmeA in DiI recruitment by assaying DiI localization in cells infected with our null strain deficient for TmeA. TmeA-deficient inclusions frequently failed to display rim-like DiI signal (Fig. 4A). Instead, DiI-containing vesicles appeared dispersed in the cytosol in a pattern that did not overlap with IncG-specific inclusion membrane staining. Quantification of DiI localization revealed a significant decrease in, but not complete ablation of, DiI co-localization with the inclusion membrane in the absence of TmeA (Fig. 4B). The defect was fully restored to WT levels in the cis-complemented *tmeA* strain. We tested whether the TmeA-dependent effect was specific to DiI by comparing NBD-ceramide trafficking to WT and Δ*tmeA* inclusions. At 24 h post-infection, accumulation of NBD-ceramide by Δ*tmeA* inclusions was comparable to WT, whereas a control treatment of WT-infected cells with BfA significantly decreased inclusion co-localization (Fig. 4C). Collectively, these data provide functional evidence for late-cycle TmeA secretion and reveal directional trafficking of plasma membrane-derived lipid to the chlamydial inclusion that differs from a previously characterized pathway mediating sphingomyelin transfer.

### Assessing TmeA functions in DiI trafficking

**Fig 5:**
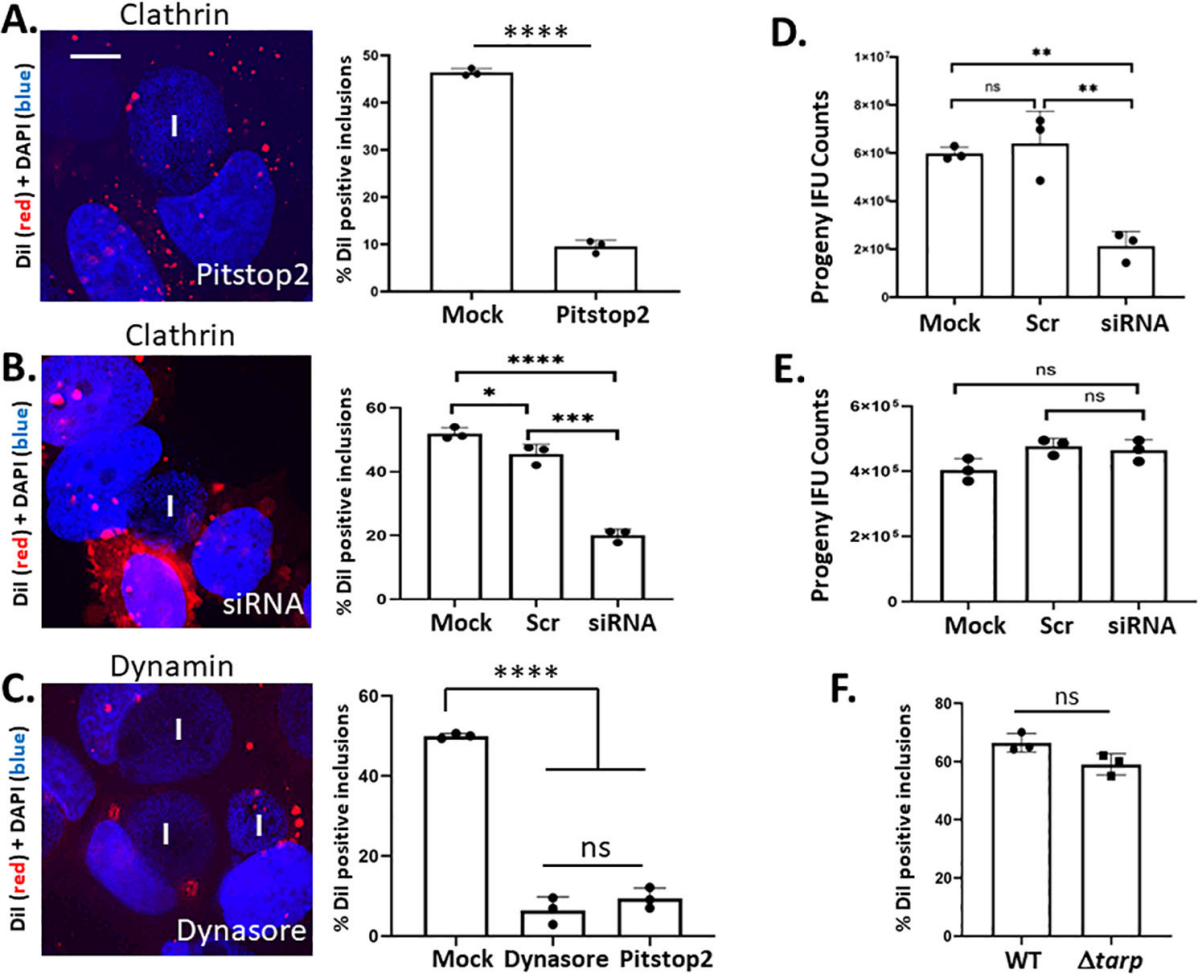
Clathrin is required for DiI trafficking to the chlamydial inclusion. HeLa cells were infected with WT L2 and cultured for 24 h. (**A**) Clathrin-dependent endocytosis was disrupted by treatment with 40 µM Pitstop 2 1 h prior and during DiI pulse. Samples were paraformaldehyde fixed after 120 min. For siRNA treatments (**B**), HeLa cultures were mock treated or transfected with clathrin-specific siRNA or scramble (Scr) control 24 h prior to infection with *C. trachomatis* L2. DiI labeling was carried out 24 h post-infection. (**C**) Dynamin or clathrin-dependent endocytosis was disrupted by treatment with 30 µM Dynasore or 40 µM Pitstop 2, respectively. Representative images showing DiI (red) or DAPI (blue) signal are shown from each experimental treatment, and inclusion (I) position is indicated. The presence or absence of DiI inclusion staining in 100 inclusions was assessed for quantitation comparing mock and inhibitor-treated cultures. Quantitative values are represented as overall (bars) and individual (closed circles) averages from triplicate cultures with SD. Statistical significance was assessed by Student’s *T* test when comparing two treatments or one-way ANOVA with multiple comparisons for >2 conditions (ns = not significant; *, *P* < 0.01; ***, *P* < 0.001; ****, *P* < 0.0001). (**D and E**) HeLa cultures were mock treated or transfected with clathrin-specific siRNA or scramble (Scr) control 24 h prior to infection with WT (**D**) or Δ*tmeA* (**E**) *C. trachomatis*. IFU-normalized material was passaged onto fresh HeLa for progeny EB enumeration at 24 h post-infection. Data are represented as means of triplicate samples (closed circles) with SDs. Statistical significance was determined by one-way ANOVA with multiple comparisons (ns = not significant, **, *P* < 0.01). (**F**) HeLa cells were infected with WT or Δ*tarp* for 24 h. Quantitation of DiI co-localization with inclusions (*n* = 100) was determined 120 min after DiI pulse in 24 h infected cultures. Data are presented as means of triplicate samples (closed circles) with SD, and a Student’s *t*-test was used for significance (ns = not significant).

We next sought to test which TmeA function(s) contribute to DiI mobilization. TmeA can promote dynamin oligomerization during clathrin-mediated chlamydial invasion (32), raising the possibility that this function may contribute during trafficking of DiI to the inclusion. We tested for a requirement of clathrin by treating cells with 40 µM Pitstop 2 prior to and during DiI pulse (Fig. 5A). Pitstop 2 treatment resulted in a significant decrease in DiI inclusion localization. In most instances, a punctate signal was detected within the host cytoplasm but not in association with 24 h inclusions. Clathrin-specific siRNA was used next to exclude the possibility of spurious inhibitor effects interfering with trafficking (Fig. 5B). Efficient knockdown of clathrin was confirmed via immunoblot (Fig. S5A). As with Pitstop 2, loss of clathrin significantly interfered with DiI co-localization with inclusions. Levels of inclusion-localized DiI in the scrambled siRNA control mirrored those of mock-treated cultures. We also tested dynamin inhibition since this protein is required for scission of invaginated clathrin pits (36). Inclusion of 30 µM Dynasore 1 h prior to and during DiI labeling (Fig. 5C) significantly decreased DiI-positive inclusions to a level similar to that observed during treatment with Pitstop 2. Neither Pitstop 2 nor Dynasore treatment prevented initial intercalation of DiI into host plasma membranes (Fig. S5C). The impact of clathrin loss on chlamydial fitness was tested to gage the importance of this pathway in chlamydial development. WT progeny EBs were enumerated from 24 h cultures that were mock-treated or transfected with siRNA 24 h prior to infection. Progeny infectious-forming units (IFUs) were enumerated from equal numbers of primary inclusions onto fresh, untreated HeLa monolayers. EBs were decreased in the presence of clathrin-specific siRNA compared to mock-treated or control siRNA-treated cultures (Fig. 5D), indicating that clathrin supports chlamydial growth. We assessed whether clathrin inhibition further impacted Δ*tmeA* growth to test whether decreased fitness phenotypes exhibited during clathrin loss or TmeA deficiency are linked. Treatment with siRNA failed to manifest a decrease in Δ*tmeA* progeny (Fig. 5E), a result that is consistent with TmeA and clathrin functioning in the same pathway. Finally, we tested whether Tarp was required for DiI trafficking since the TmeA-dependent dynamin oligomerization requires prior action by Tarp (32). Rim-like staining of inclusions with DiI was not affected during infection with Δ*tarp* (Fig. 5F), suggesting that TmeA is not directly acting on dynamin and clathrin to promote DiI mobilization. Furthermore, co-localization of inclusions with the transferrin receptor has been reported to require clathrin-mediated endocytosis (37). Loss of TmeA did not impact accumulation of peri-inclusion transferrin receptor (Fig. S5B), indicating specificity for TmeA and clathrin in DiI trafficking. In aggregate, these data indicate that TmeA and clathrin reside in the same pathway but perform separate functions in supporting DiI accumulation by inclusions. Furthermore, this pathway is important for the intracellular development of chlamydiae.

**Fig 6:**
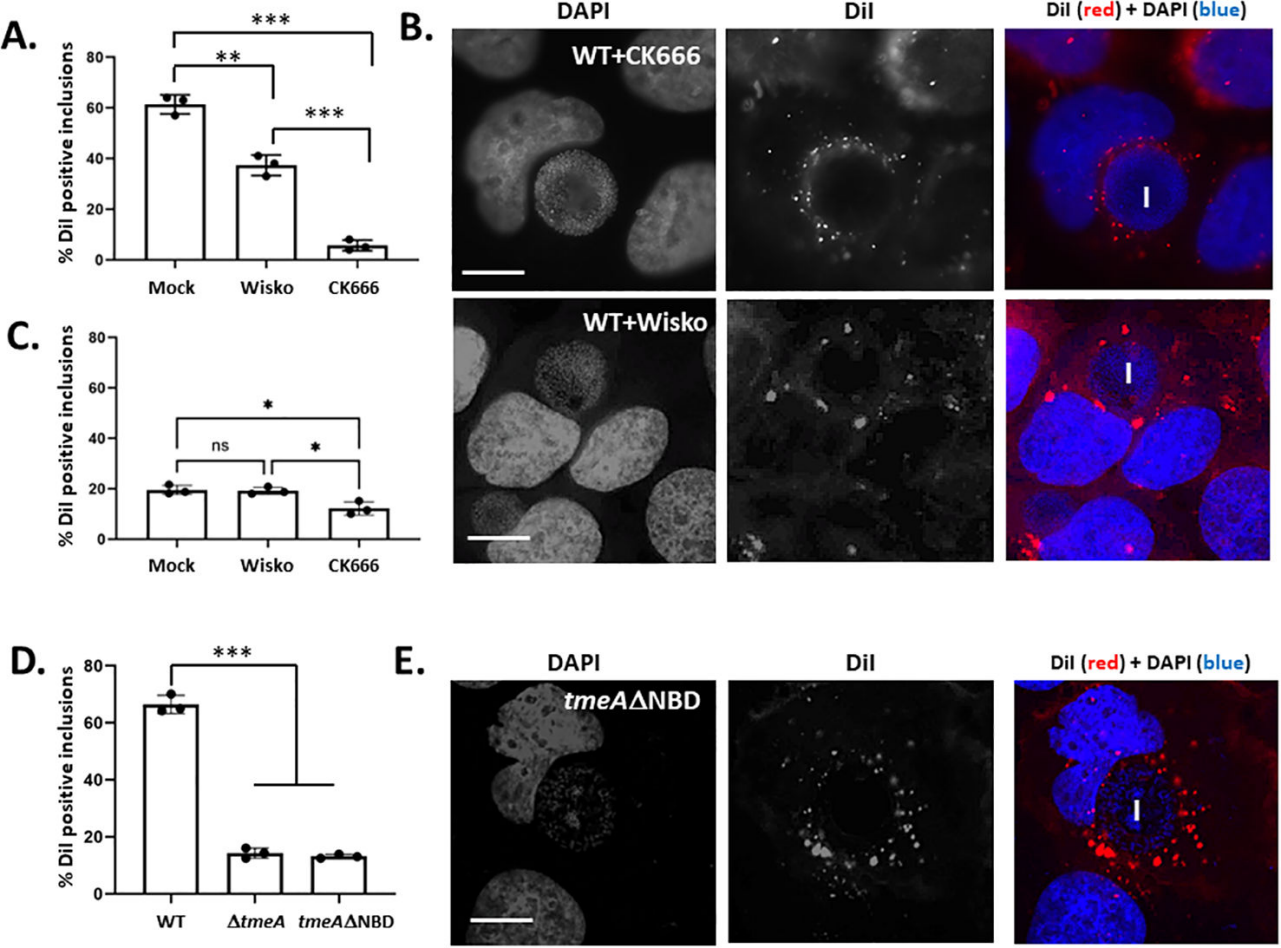
TmeA N-WASP-activating activity is required for delivery of surface-localized DiI to the chlamydial inclusion. (**A**) Quantitation of DiI co-localization with inclusions (*n* = 100) after 120 min in HeLa cultures infected with WT for 24 h. Cultures were untreated (mock) or treated with 7.5 µM Wiskostatin (wisko) or 200 µM CK666 30 min prior to and during DiI pulse. (**B**) Representative images of Wiskostatin and CK666-treated cultures. Cultures were stained 120 min after DiI pulse and DiI (red) or DAPI (blue) signals are shown. Inclusion (I) position is indicated in the merged images. Bar = 10 µm. (**C**) Quantitation of DiI co-localization with Δ*tmeA* 24 h inclusions (*n* = 100) where cultures were untreated or treated with 7.5 µM Wiskostatin (wisko) or 200 µM CK666 30 min prior to DiI pulse. (**D**) Inclusions (*n* = 100) were scored for localization of DiI with WT, Δ*tmeA*, or *tmeA*-ΔNBD inclusions at 24 h post infection. (**E**) Representative fluorescence images of HeLa cells infected for 24 h *tmeA*ΔNBD and fixed 120 min after DiI pulse. DiI (red) or DAPI (blue) signals are shown with inclusion (I) position indicated in the merged images. All bar = 10 µm. All quantitative data are represented as means of triplicate samples (closed circles) with corresponding SDs. Statistical significance was determined by one-way ANOVA with multiple comparisons (ns = not significant, *, *P* < 0.01; **, *P* < 0.001; ***, *P* < 0.0001).

TmeA promotes Arp2/3-dependent branched actin polymerization by binding (31) and activating (25) N-WASP during invasion. Arp2/3-mediated actin polymerization can provide a scaffold for plasma membrane-derived vesicle migration and generate motive force driving vesicular migration (38, 39), raising the possibility that the actin polymerization-inducing function of TmeA could contribute to DiI trafficking to the inclusion. TmeA-mediated stimulation of actin polymerization requires association with N-WASP, and TmeA is capable of associating with N-WASP during late-cycle development (25). We began by testing the possibility of TmeA-dependent N-WASP activation in DiI trafficking using pharmacological inhibition. WT-infected HeLa cultures were pulsed with DiI in the presence of the N-WASP inhibitor Wiskostatin, the Arp2/3 inhibitor CK666, or vehicle control. Inclusion membrane staining with DiI was visually quantified after 120 min (Fig. 6A). Treatment with 7.5 µM Wiskostatin interfered with DiI co-localization compared to the untreated control, yet the magnitude of inhibition was not as robust as that achieved by 200 µM CK666 treatment. DiI-containing vesicles appeared dispersed within the host cytosol in the presence of either inhibitor (Fig. 6B). These experiments were repeated using *DtmeA* infection (Fig. 6C). Treatment of cultures with Wiskostatin did not decrease the abundance of DiI-positive inclusions, whereas CK666 treatment enhanced the *DtmeA* defect by approximately twofold. These results suggest that TmeA-dependent DiI inclusion co-localization manifests via N-WASP activation and implicates additional roles for Arp2/3.

We next constructed a strain expressing TmeA lacking residues 118–126, representing the minimal NBD to test this finding more directly. Allelic replacement was used to restore *tmeA*-ΔNBD to our Δ*tmeA* strain. Immunoblot-verified expression of TmeA-ΔNBD (Fig. S6A) and, as expected, invasion assays confirmed that loss of the NBD interfered with invasion efficiency to a level similar to that manifested by the null *tmeA* strain (Fig. S6B). Intracellular development in the presence of TmeA-ΔNBD correlated with a loss of fitness (Fig. S6C). DiI labeling was examined in HeLa cells equally infected with WT, Δ*tmeA*, or *tmeA*-ΔNBD at 24 h. Quantification indicated a loss of DiI co-localization with inclusions similar to that detected in the null *tmeA* stain (Fig. 6D). These data indicate that TmeA-induced actin polymerization via N-WASP plays a significant role in the accumulation of plasma membrane-derived material by inclusions.

### TmeA co-localization with DiI

**Fig 7:**
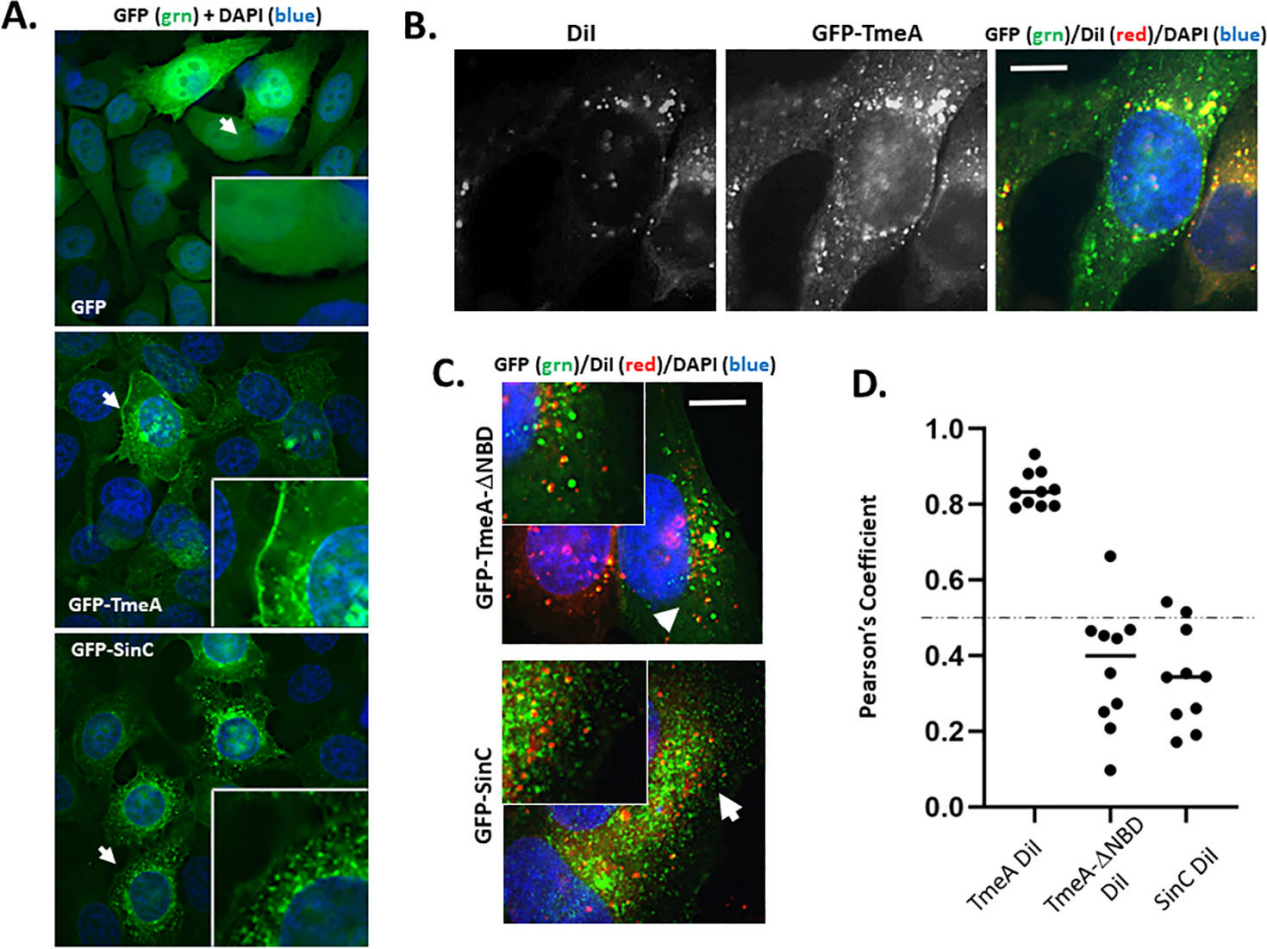
Ectopically expressed TmeA co-localizes with internalized DiI. (**A**) Epifluorescence images of uninfected HeLa cells ectopically expressing GFP, GFP-TmeA, or GFP-SINC for 24 h. Nuclei were detected after fixation with DAPI (blue). Arrows indicate area of inset and bar = 10 µm. (**B**) HeLa cells expressing GFP-TmeA were pulsed with DiI and incubated for 90 min followed by paraformaldehyde fixation. Localization was visualized by direct fluorescence, and representative images are shown. (**C**) GFP-TmeAΔNBD or GFP-SINC expressing HeLa cells were pulsed with DiI (red) and fixed for visualization at 90 min. Arrows indicate area of inset in merged images. Bars = 10 µm. (**D**) Corresponding Pearson co-localization coefficients of respective signals were calculated from 10 inclusions. The dashed line indicates a 0.5 coefficient cutoff.

TmeA was not detected in the inclusion membrane, yet the staining pattern manifested by secreted TmeA-Flag suggests that TmeA and DiI could co-localize in vesicles prior to delivery of DiI to inclusion membranes. Moreover, expression of TmeA-Flag starting during mid-cycle (12 h) restored the ability of tmeA to recruit DiI (Fig. S7). Unfortunately, we were unable to visualize TmeA-Flag using saponin permeabilization required to prevent interference with DiI localization. However, TmeA-GFP localizes in peri-nuclear punctate foci when ectopically expressed in HeLa cells (26). We returned to ectopic expression studies to address whether TmeA alone co-localizes with internalized DiI. In contrast to GFP alone, GFP-tagged TmeA accumulated at the cell periphery as previously observed (26), and in punctate, vesicular-like structures distributed within the host cytosol (Fig. 7A). GFP-TmeA did not overlap with the Golgi apparatus (Fig. S8A), indicating that the vesicular structures were not part of the Golgi. *Chlamydia caviae* CCA0062 (designated SinC) is encoded at the same genomic locus of TmeA, but the two proteins are not homologous (23, 40). We chose to use this protein as a control since GFP-SinC functions at the nuclear membrane (41). In addition, *C. caviae* inclusions are morphologically distinct from those of *C. trachomatis* (42), and *C. caviae* inclusions did not accumulate DiI signal (Fig. S8B). Expression of GFP-SinC exhibited peri-nuclear concentration in punctate structures. We tested whether TmeA co-localized with DiI by pulsing HeLa cells expressing GFP-TmeA with DiI. Punctate DiI-specific signal overlapped frequently with GFP-TmeA (Fig. 7B). This was specific for DiI since fluorescent transferrin internalized by TmeA-expressing cells did not overlap (Fig. S8C). In contrast, similar treatment of cells expressing either GFP-TmeA-NBD or GFP-SinC failed to exhibit the same co-localization of DiI signal (Fig. 7C). Calculation of Pearson coefficients confirmed significant overlap of DiI signal with GFP-TmeA but not GFP-TmeA-NBD or the negative control GFP-SinC (Fig. 7D). Regardless, punctate intracellular patterns were still detected for GFP-TmeA-NBD (Fig. S8D), indicating that interaction with N-WASP is not required for these structures. These data indicate that TmeA alone can co-localize with internalized DiI-containing vesicles, and this requires the N-WASP binding domain of TmeA. The interaction is specific since the functionally divergent SinC control failed to similarly co-localize with internalized DiI, and TmeA does not co-localize with transferrin-containing vesicles.

## DISCUSSION

TmeA was formerly characterized solely as an invasion-related effector secreted from EBs upon host cell attachment (23), where it contributes to host cell entry (24). After invasion, *de novo* synthesis of TmeA only occurs during late cycle development (21). Our data confirm late-cycle production of TmeA during our handling. This pool of TmeA was originally postulated to be packaged only into newly forming EBs for subsequent rounds of infection (23). Subsequent data suggested an additional destination. BlaM (33) and split GFP (34) reporter studies have shown that ectopically expressed TmeA can be secreted by RBs in mature inclusions. In addition, proximity labeling experiments indicated that ectopically expressed TmeA can interact with N-WASP during intracellular development (25). Our new findings provide direct evidence that TmeA functions after invasion to facilitate intracellular development. First, chlamydiae lacking *tmeA* form smaller inclusions that result in a modest, but significant, decrease in progeny EB production. Second, our data provide additional evidence that TmeA is secreted from mature inclusions. *Chlamydia*-localized TmeA is maintained in a soluble state via the chaperone Slc1 (43), whereas secreted TmeA peripherally associates with host plasma membranes through a discrete helical domain similar to *Yersinia* YopE and *Pseudomonas* ExoS effectors (26). We were able to use Triton X114 partitioning to confirm for the first time that endogenous TmeA is secreted from mature inclusions. In addition, Flag-tagged TmeA was detected within the host cytosol. Finally, the requirement for TmeA in DiI trafficking to mature inclusions provides direct indications for late-cycle function.

**Fig 8:**
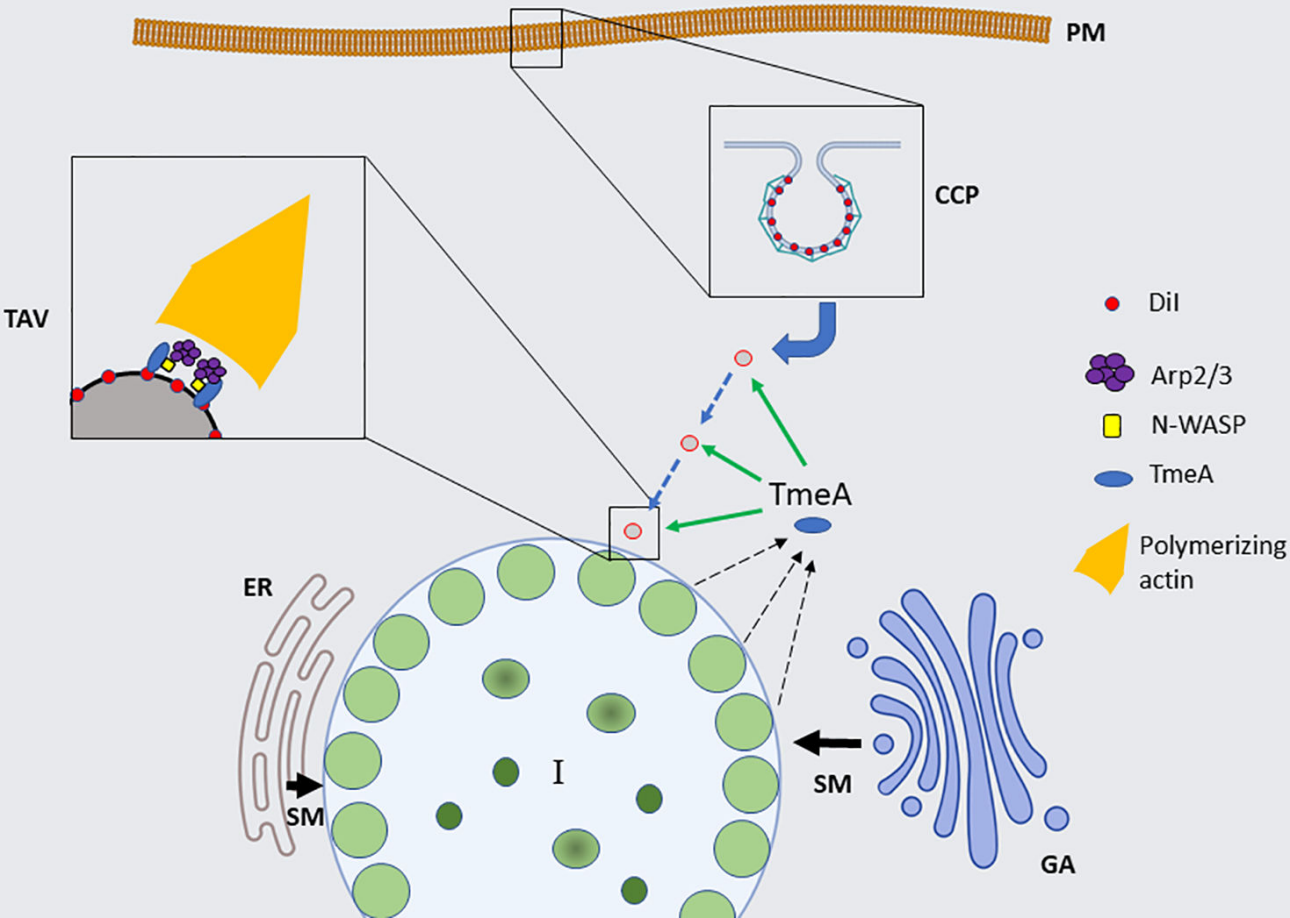
Working model of TmeA-dependent DiI trafficking. Internalization of material from the host plasma membrane (PM) is accomplished by the formation of clathrin-coated pits (CCP) resulting in endocytosis. TmeA is secreted (black arrows) from mature inclusions (I) where it can associate with discrete subsets of vesicles. Subsequent association of TmeA with N-WASP stimulates local actin polymerization, providing motility that directs TmeA-associated vesicles (TAV) to the inclusion, where subsequent events promote vesicle fusion with the IM. This mechanism is distinct from pathways that result in sphingomyelin (SM) recruitment from organelles such as the Golgi apparatus (GA) or ER.

We favor a working model where TmeA is involved in the intracellular trafficking of DiI-laden vesicles (Fig. 8). The localization of TmeA-Flag and membrane partitioning of endogenous TmeA would be consistent with an association with intracellular vesicles. The observation that GFP-TmeA co-localizes with DiI-containing vacuoles is also consistent with secreted TmeA being involved in vesicular trafficking. We regard it as unlikely that carry-over of TmeA secreted during invasion could explain any of these observations. TmeA is part of a highly redundant arsenal of proteins involved in chlamydial entry (3), in which the loss of one factor has modest and transient effects on entry. Our data indicate that any invasion defect manifested in the absence of TmeA does not directly translate to decreased intracellular growth. Intuitively, it is difficult to envision how a small pool of effector could mediate robust redirection of plasma membrane-derived material to the inclusion. In addition, TmeA message and protein were below detection at 12 h post-infection and induction of ectopic TmeA-Flag after invasion supported DiI recruitment. The repurposing of an early-acting effector makes sense. *Chlamydia* possesses a highly reduced genome (44), and reliance on effectors to accomplish roles during multiple developmental stages improves efficiency of coding capacity.

The mechanism by which TmeA mediates DiI trafficking represents an intriguing area. TmeA interacts with host AHNAK (23), but infection of AHNAK-deficient cells does not affect invasion or intracellular growth (24). Instead, data implicate both clathrin/dynamin-2 and actin polymerization. Interference with clathrin-mediated endocytosis decreases chlamydial entry (45). TmeA and Tarp have been implicated in the activation of Dynamin-2-mediated scission of *Chlamydia*-containing vesicles from the plasma membrane during invasion (32), raising the possibility that TmeA could be important for the initial steps of clathrin-mediated endocytosis of DiI-containing material. However, this TmeA activity requires initial contributions from TarP, and the Δ*tarp* strain did not affect IM staining with DiI. Regardless, clathrin and TmeA appear to act in the same pathway since knockdown of clathrin decreased WT but not Δ*tmeA* progeny. Clathrin contributes to a wide spectrum of physiological functions ranging from endocytosis to vesicular trafficking (46, 47). Our working model (Fig. 8) posits that clathrin functions upstream of TmeA by mediating endocytosis of DiI-containing material. In support of this notion, DiI-containing vesicles still appeared to be internalized in the absence of TmeA, indicating that TmeA is not required for this process. Furthermore, *Ehrlichia* appear to attain lipids via bulk transfer from the plasma membrane after clathrin-mediated endocytosis (35).

TmeA-mediated activation of actin polymerization via N-WASP was directly involved in DiI localization to IMs. Pharmacologic inhibition of either N-WASP or Arp2/3 interfered with DiI labeling of inclusions. Most significantly, DiI IM labeling was absent when cells were infected with chlamydiae expressing TmeA lacking the N-WASP binding domain. Both N-WASP and Arp2/3 can function in vesicle scission during endocytosis (48). However, actin polymerization only appears relevant for vesicles containing large cargos (38). Internalized DiI vesicles were also apparent in the presence of N-WASP or Arp2/3 inhibition, and ectopically expressed NBD-deficient TmeA failed to co-localize with DiI vesicles. We, therefore, favor a function for TmeA-mediated actin polymerization after endocytic events at the plasma membrane. TmeA was recently shown to interact, via an undefined mechanism, with the transducer of Cdc42-dependent actin assembly (TOCA-1) (49). We did not test for a requirement of TOCA-1 since this protein functions through N-WASP.

While Arp2/3-dependent actin polymerization is established as an important player in chlamydial entry and exit (3), a requirement during intracellular development has not been previously described. F-actin has emerged as a factor governing the sustained integrity of the intracellular compartment for pathogens residing in a parasitophorous vacuole. Prior to vacuolar escape, Arp2/3 mediates actin polymerization around *Shigella*-containing vacuoles, and inhibition of this actin cocoon by CK666 impairs vacuolar escape (50). Mature *Chlamydia* inclusions are also encased in dynamic cages of polymerized actin that provide a structural scaffold promoting inclusion stability (51). These structures, however, are not essential for growth and require the RhoA GTPase (52) but not Arp2/3 activity for formation (53). Interestingly, F-actin patches localize to developing *Coxiella*-containing vacuoles (CCV) where they provide sites for Rab7-dependent fusion with late endosomal vesicles (54). In *Coxiella*, CK666 significantly reduced the intracellular fitness of *Coxiella burnetii*. Arp2/3 activity was found to support growth through trafficking of endocytic vesicles to the CCV (54). This pathway is unlikely to be relevant for chlamydiae, since fusion with the endolysosomal compartment is actively blocked (3), and *Chlamydia* interferes with general retrograde transport (55). F-actin polymerization via Arp2/3 provides forces supporting membrane dynamics at numerous sites. These include well-established cytoplasmic roles in endosome sorting, vesicle motility, and release of vesicles from the *trans*-Golgi network (48). N-WASP is predominantly associated with Golgi-ER transport and vesicle motility (56). We observed that DiI trafficking was BfA insensitive, and loss of TmeA did not impact SM trafficking from the Golgi. We therefore favor a role for TmeA in vesicle motility (Fig. 8), in which F-actin polymerization supports the propulsion of internalized vesicles within the cytosol. Our data showing GFP-TmeA and DiI-containing material concentrated toward the host nucleus supports this notion, and future work is directed at testing this hypothesis.

The presence of additional lipid transfer pathways is perhaps not surprising given the importance of host resources to support chlamydial infection. We directly tested whether the chlamydial inclusion directly parasitizes the plasma membrane using the lipophilic dye DiI. DiI is non-toxic, does not passively transfer among membranes, and has been used to trace trafficking pathways (57–59). For example, DiI was recently used to demonstrate unidirectional trafficking of plasma-membrane-derived lipids via an autophagic mechanism to intracellular *Ehrlichia* (35). Hackstadt et al. specifically excluded the possibility of the host plasma membrane as a direct source of NBD ceramide (10). These studies relied on visualization of individual lipids, raising the possibility that abundance issues could have confounded detection. Alternatively, chlamydial capture of DiI tracks discrete plasma membrane content. Regardless, the work presented herein indicates a pathway for DiI trafficking that is distinct from sphingomyelin acquisition.

Indeed, *Chlamydia* appears to be selective in the recruitment of material from the plasma membrane via this pathway. We found that *Chlamydia*-mediated DiI, but not transferrin, trafficking required TmeA. Ectopically expressed TmeA also co-localized with DiI, but not transferrin, containing vesicles. This is significant since clathrin-mediated endocytosis of transferrin receptor results in co-localization of Tfn with chlamydial inclusions (37). These data implicate a degree of cargo selectivity. Potential roles for trafficked lipids are unclear. We observed an apparent DiI signal within the inclusion lumen, but this did not appear to coincide with individual bacteria. This localization was sporadic, and we cannot currently discriminate whether this could be a fixation artifact (60) or represent bona fide migration into the inclusion. The absence of DiI in bacteria suggests that the DiI-containing lipid pool is not directly required for chlamydial replication. Exclusion of the dye by bacteria is unlikely since *Ehrlichia* envelopes readily incorporate DiI (35). DiI signal was instead prominently detected during *Chlamydia* infection in co-localization with the IM marker IncG, raising the possibility that this pathway supplies lipids to support the expanding inclusion. Smaller inclusions manifested by the Δ*tmeA* strain would be consistent with this hypothesis. We cannot, however, exclude the possibility that DiI-containing material accumulates adjacent to the inclusion and is not incorporated directly into the IM. Further work will be required to address the specific role of DiI-indicated lipid transfer.

In aggregate, our data support a functional role of TmeA after invasion. The data also implicate a newly appreciated pathway for *Chlamydia* to parasitize host cells and support a working model (Fig. 8) where material endocytosed from the plasma membrane is redirected to the chlamydial inclusion. We speculate that a secreted pool of TmeA is deployed from the mature inclusion and associates with internalized vesicles that have acquired N-WASP. TmeA-driven activation of N-WASP results in vesicle rocketing that prevents normal recycling or endolysosomal fusion. Alternatively, TmeA may maintain vesicles at the peri-inclusion area where they can fuse with the inclusion membrane. Fusion events likely involve host machinery recruited to the chlamydial inclusion or specific Incs that mimic their function. Interestingly, the *tmeA* locus is divergent among chlamydial species, with the corresponding genes in *C. caviae* and *C. pneumoniae* encoding proteins that lack homology (23) to TmeA and appear functionally distinct (40, 41). *C. caviae* did not accumulate DiI signal, and it will be interesting to test whether this pathway is manifested in other chlamydial species.

## MATERIALS AND METHODS

### Organisms and cell culture

*C. trachomatis* serovar L2 (LGV 434) was used as the parent, wild-type strain in these studies. Previously described strains include *C. trachomatis* D*tarp* (61), *tmeA-lx* ([25]; referred to herein simply as Δ*tmeA*), and cis-complemented *tmeA* (cis-*tmeA* [25]). *C. trachomatis* lacking the *tmeA*-encoded N-WASP binding domain (*tmeA-*ΔNBD) was generated using the same allelic replacement strategy used to generate cis-tmeA from Δ*tmeA* (25) according to established FRAEM protocols (62, 63). In this instance, the *tmeA* sequence lacked the codons for residues 118–126 required for N-WASP (31). Inducible expression of Flag-tagged TmeA was accomplished by PCR-based (Table S1) insertion of full-length *tmeA* tagged with a 3’ Flag-encoding sequence downstream of *tetO* in pBOMB4 (64). *C. trachomatis* expressing pBOMB-NrdB-FT was described previously (64). All chlamydial transformations were accomplished using CaCl_2_-mediated chemical transformation (65), and clonal isolates for all final *Chlamydia* strains were obtained by limiting dilution in 384 plates as described (62). *C. caviae* (GPIC) was also used as a control species and cultivated similarly to *C. trachomatis*. Chlamydiae were routinely maintained in McCoy (CRL-1696; ATCC) cell cultures for genetic manipulations. Experiments were carried out as indicated in either HeLa 229 epithelial cell monolayers (CCL-1.2; ATCC) or A2EN (66) endocervical epithelial cultures. Unless otherwise indicated, McCoy and HeLa were grown in RPMI 1640 medium containing 2 mM L-glutamine (Life Technologies) supplemented with 10% (vol/vol) heat-inactivated fetal bovine serum (Sigma). A2EN were cultivated in Gibco KSFM media supplemented as described (66). All cultures were maintained at 37°C in an environment with 5% CO_2_ and 95% humidified air. Infections were accomplished by centrifugation of EBs onto cell monolayers at 20°C for 1 h at 900 × g or rocking on ice when appropriate for invasion assays. Where indicated, infected cultures were supplemented with Brefeldin A (Invitrogen), Wiskostatin (Sigma), CK666 (Sigma), Dynasore (Sigma), or Pitstop-2 (Sigma) at indicated concentrations to interfere with target protein activity. Cultures were supplemented with aTc (Sigma) for induced expression studies. For knockdown experiments, HeLa monolayers were transfected with clathrin-specific siRNA or scrambled control siRNA (ThermoFisher) using RNAiMax (Life Technologies) 24 h prior to infection.

### Immunodetection and microscopy

For immunoblot analyses, proteins were separated on 4%–15% SDS-PAGE gels (Bio-Rad) and transferred to 0.45 µm PVDF membranes (Millipore). Primary antibodies were specific for: TmeA (23), TmeB (33), Hsp60 (A57-B9, Santa Cruz), MOMP (23), Flag-M2 (Sigma), clathrin (Santa Cruz), or GAPDH (Cell Signaling). Peroxidase-conjugated secondary antibodies (Sigma) and Amersham ECL Plus (GE Healthcare UK Limited) detection reagents were used to visualize proteins. Detection via fluorescence microscopy was accomplished using direct fluorescence of paraformaldehyde-fixed HeLa cultures probed with Alexa488-Transferrin (Invitrogen) or 1,1′-dioctadecyl-3,3,3',3′-tetramethylindocarbocyanine perchlorate (DiI; Invitrogen) or for cells expressing GFP-TmeA, NBD-deficient GFP-TmeA, or mCherry-TmeA (23). GFP-SinC was constructed by PCR amplification using custom primers (Table S1) specific for full-length *cca00062* from *C. caviae* and inserted in-frame with GFP expressed from eGFP-C3, similarly to TmeA constructs. For indirect immunofluorescence, cultures were fixed with 4% paraformaldehyde, and chlamydiae were routinely visualized by DAPI staining. Permeabilization was accomplished where required 0.1% Triton X100, except for experiments including visualizing DiI and IncG localization, where permeabilization was accomplished with 0.05% saponin. Detection of additional proteins was accomplished using antibodies specific for: IncG (67), Transferrin Receptor (Invitrogen), Flag-M2, or GM130 (BD Transduction). Visualization was accomplished using secondary antibodies conjugated to AlexaFluor-594 or -488 (Invitrogen). Cells were examined via epifluorescence microscopy using a Nikon E800 Eclipse with 100× oil immersion objective or via confocal using a Nikon A1R inverted confocal microscope with 63× oil immersion objective. All images were processed equivalently using auto contrast and unsharp mask filter functions in Adobe Photoshop 6.0 (Adobe Systems). Images were imported into ImageJ (68) for the generation of signal intensity plots and calculation of signal overlap for the generation of Pearson coefficients.

### Fitness assays

For invasion assays, HeLa cells were prepared in 24-well plates with 12 mm coverslips, and invasion assays were performed essentially as described (69) using density gradient purified EBs at a multiplicity of infection (MOI) of 20. Briefly, infections were carried out on ice with rocking for 1 h then shifted to 37°C for 30 min. Cultures were fixed with 4% paraformaldehyde, and extracellular or intracellular EBs were differentially labeled with murine lipopolysaccharide (LPS)-specific or rabbit MOMP antibodies, respectively. Detection was accomplished with a secondary antibody conjugated to Alexa-594 (anti-mouse) or Alexa-488 (anti-rabbit). Percentages of invaded chlamydiae were computed by enumeration of internal and external chlamydiae in 10 fields of view. Percent EB internalization was calculated via the formula ((total EBs – external EBs)/total red EBs) × 100 = percent (%) invasion. These experiments were performed three times. Chlamydial growth was evaluated by inclusion area calculation in primary cultures or progeny EB enumeration by passage onto secondary cultures. Inclusion areas were calculated from 1,000 random inclusion images acquired from methanol-fix inclusions visualized by Hsp60 staining. For progeny assays, cultures were mechanically disrupted at 24 h post-infection. Parallel cultures were methanol fixed to assess the inclusion number in primary cultures to normalize progeny output. Inclusions derived from progeny IFU were enumerated on fresh HeLa monolayers after 24 h as described (70). For particle-normalized primary infections, serial dilutions of density gradient-purified EBs were pelleted onto glass coverslips at 900 × *g* for 60 min. Material was fixed with 4% paraformaldehyde and stained with DAPI, and EBs were enumerated by direct microscopic visualization. Input infection material for strains was performed with equal particle numbers and the process for inclusion counts as described above. All growth data were derived from triplicate replicates for each strain/condition, and experiments were performed at least three times.

### Analysis of protein secretion and temporal expression

Membrane partitioning was used as described (26) to evaluate TmeA secretion. Lysates from density-gradient purified EBs or from 24 h whole-culture material were extracted with 1.0% Triton X114 as described (26). trichloroacetic acid (TCA) concentrated material was analyzed by immunoblot using specific antibodies. For temporal expression, HeLa cultures were infected at an MOI of 1 and protein or RNA harvested at 12 h, 16 h, or 24 h post-infection. Proteins were detected via immunoblot. For assessment of gene expression, the Aurum Total RNA minikit (Bio Rad) was used to isolate RNA. Subsequent generation of cDNA and gene amplification was achieved using iTaq Universal SYBR Green One-Step kit (Bio Rad) and gene-specific primers (Table S1) as described (71).

### Co-localization studies

Trafficking of Golgi-derived lipids to the chlamydial inclusion was carried out using BODIPY FL C5-Ceramide (N-(4,4-difluoro-5,7-dimethyl-4-bora-3a,4a-diaza-s-indacene-3-pentanoyl)Sphingosine) as described (10). Briefly, 24 h infected cultures grown on glass coverslips were pulsed with 5 µM Bodipy-ceramide in RPMI supplemented with 0.034% defatted bovine serum albumin for 30 min on ice. Cultures were washed then incubated at 37°C in RPMI + 0.34% dfBSA for 90 min. Live cells were visualized via epifluorescence and bright-field microscopy to identify inclusions, and 100 cells per replicate were examined and visually scored for the presence or absence of intra-inclusion staining. Where indicated, cultures were treated with 3 µg/mL Brefeldin A for 1 h prior to and during labeling. Vybrant DiI Cell-Labeling Solution (Invitrogen) was used to assess trafficking from the plasma membrane. *Chlamydia*-infected cells cultivated on 12 mm glass coverslips were pulsed with 5 µM DiI for 5 min on ice, washed three times, and incubated for various times at 37°C. Cultures were fixed with 4% paraformaldehyde and counterstained with DAPI or IncG-specific antibodies. Coverslips were mounted with solvent-deficient ProLong Diamond (Invitrogen) mounting medium and examined via fluorescence microscopy. Cells were scored for the presence or absence of rim-like inclusion-membrane localized material encompassing the complete inclusion boundary and values indicated as “percent DiI positive inclusions.” For co-localization of DiI or Transferrin with ectopically expressed tagged proteins, HeLa cells were nucleofected (Lonza) with respective plasmid DNAs and pulsed with DiI or Alexa-conjugated Transferrin (Invitrogen) 24 h later. For Transferrin, cells were pulsed for 4 h with 2.5 µg Transferrin, washed, paraformaldehyde fixed, and visualized by direct fluorescence. Images were imported into ImageJ to generate fluorescence intensity profiles and for quantitative assessment of signal overlap. For ectopic expression studies, the overlap of signal areas was calculated for 10 cells per replicate (in pixel) to yield percent overlap values. Corresponding Pearson coefficients of fluorescence overlap were then calculated using BIOP’s version of JACoP (68). Values exceeding 0.5 were considered significant overlap of signals as reported (72).

### Statistical analysis

Unless otherwise noted, the presented data are representative results from experiments repeated at least three times. Quantitative data were generated from triplicate biological and technical replicates during each experiment. All quantitative data are presented as mean values with one SD. Statistical significance was assessed via Student’s *t*-test with Welch’s correction when comparing two data sets and via one-way ANOVA with multiple comparisons for experiments with >2 data sets. Statistical analyses were performed using GraphPad Prism 6 version 6.04 (GraphPad Software Inc).

## Data Availability

Whole-genome sequence data can be accessed at BioProject-NCBI under BioProject PRJNA1309005, BioSamples SAMN50722639 and SAMN50927177. All additional data required to assess conclusions are present in the paper or the Supplementary Materials.

## SUPPLEMENTAL MATERIAL

Supplemental Material mbio.01993-25-s0001.docx Supplemental table and figures.

ASM does not own the copyrights to Supplemental Material that may be linked to, or accessed through, an article. The authors have granted ASM a non-exclusive, world-wide license to publish the Supplemental Material files. Please contact the corresponding author directly for reuse.
