# Supplementary material for: Evidence for a post-invasion role of the Chlamydia trachomatis type III secreted effector TmeA in redirection of host plasma membrane-derived material
Source: mBio. 2025 Sep 25;16(11):e01993-25. doi: 10.1128/mbio.01993-25 (PMC12607697; doi:10.1128/mbio.01993-25)
Supplement: Supplemental Material — Supplemental table and figures. [file mbio.01993-25-s0001.docx]

| **Custom Primers** | |
| --- | --- |
| *cloning primers* | **Primer Sequence (5’→ 3’)** |
| 694FLAG-F | AGAAAGGATCTGCGGCCGCGTTGGAATTAAAAGTTATTGCTTCGGCGG |
| 694FLAG-R | GGTCGACCGGTACCTGCAGTTACTTGTCATCGTCGTCCTTGTAGTCGTCTAAGAAAACAGAAGAAGTTATGACAG |
| CCA62-F | AAACCCAAGCTTGGAATTAATCCAAGCGGTCGAAACAATAAC |
| CCA62-R | CGGGGTACCCTATTCTTCTGAACTAAAAGCATCCAAGCC |
|  |  |
| *qRT-PCR primers* |  |
| rpoD-s | GCGGTGTTTCCATTGTCGTCATA |
| rpoD-as | ATTTCTCTCAGCTCGCGCTTTC |
| tmeA-s | TAACCTATCTGTGGGAGGGAAG |
| tmeA-as | GGCATCTACCGTAGGATCTGTA |
| momp-s | CACTTGGTGTGACGCTATCA |
| momp-as | GGCACCCATCTGAAATTCTTTATT |

**Supplementary material**

**Supplementary Table 1. Custom Primers**

**
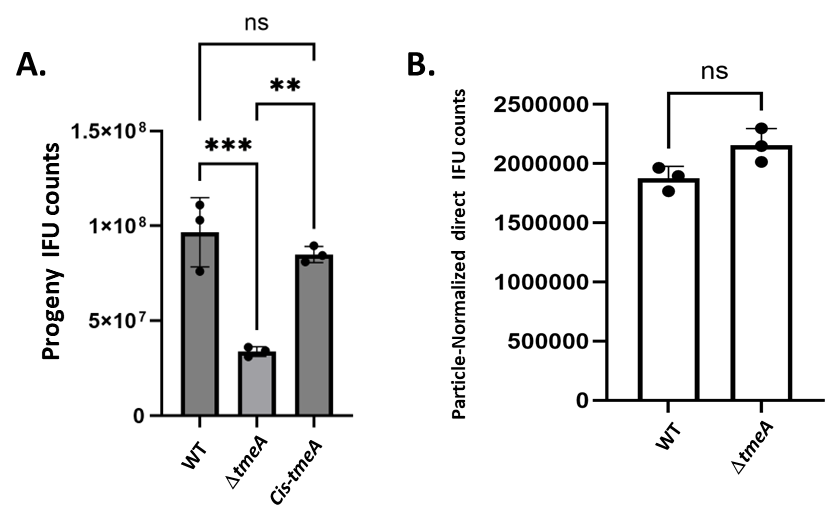
**

**Figure S1.** (A). Progeny EBs from triplicate HeLa cultures equally infected with WT, Δ*tmeA*, or cis-*tmeA* were enumerated after 48 hrs cultivation of passaged material. Progeny count data are represented as means of culture replicates (closed circles) with corresponding standard deviations. Statistical significance was determined by One-way ANOVA with multiple comparisons (ns = not significant; **, P<.003; ***, *P*<0.001). (B) HeLa cells Particle normalized WT and Δ*tmeA* infections yield similar inclusion numbers. Equivalent numbers of DAPI-positive particles from density gradient-purified WT and Δ*tmeA* EB preps were used to equally infect HeLa cells at an MOI of 0.01. Cultures were methanol fixed at 24 hrs post infection and inclusions enumerated after staining with Hsp60-specific antibodies. Data are represented as mean inclusion numbers from triplicate samples (individual averages indicated by closed circles) with one standard deviation. Statistical significance was determined by Student’s T test (ns = not significant).


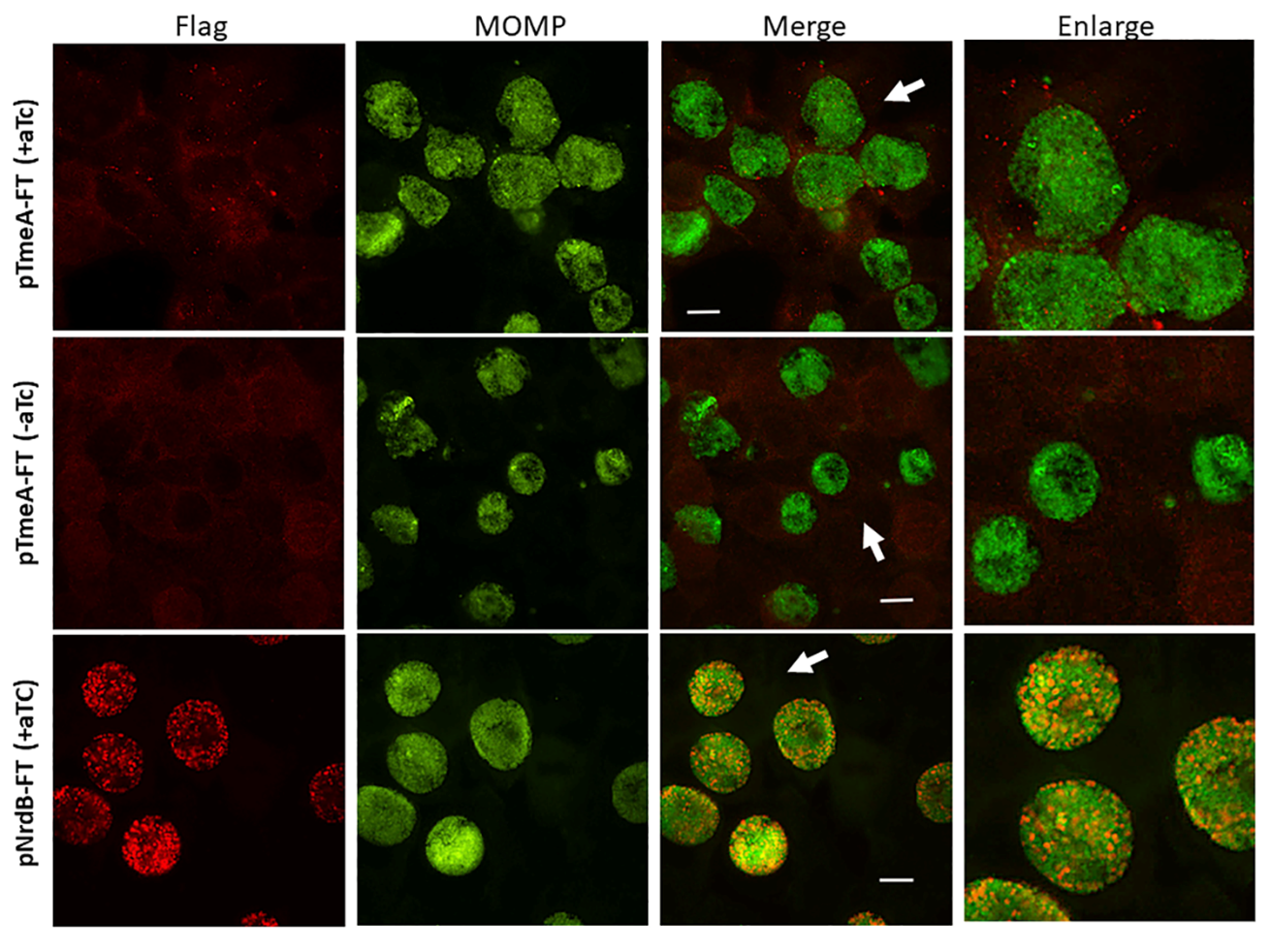


**Figure S2**. Confluent HeLa cell monolayers were infected with Δ*tmeA* expressing pBOMB-TmeA-FT or WT expressing pBOMB-NrdB-FT. Media were supplemented with inducer (+aTc) or mock treated (-aTc) at 12 hrs post infection and processed at 30 hrs post infection. Cultures were fixed with paraformaldehyde followed by ice-cold methanol. TmeA an NrdB were detected using Flag (red) whereas chlamydiae were detected using MOMP (green)-specific antibodies. Arrows indicate area of enlargement. Bar = 10 µm.

**
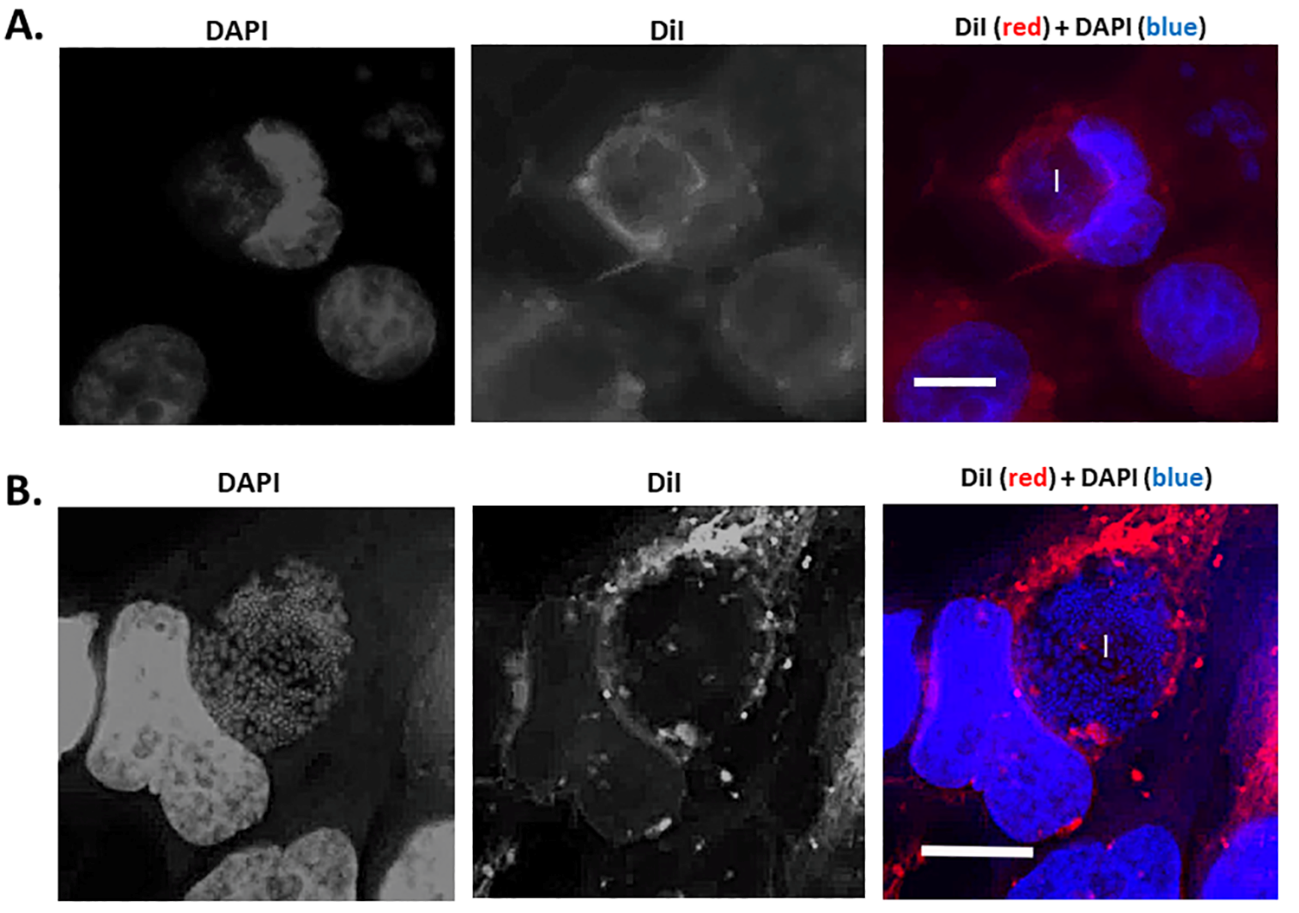
**

**Figure S3.** Epi-fluorescence images of DiI-pulsed A2EN or HeLa cultures infected with *C. trachomatis* serovar L2 for 24 hr. (A) A2EN cultures were paraformaldehyde fixed at 120 min after DiI pulse and counterstained with DAPI to visualize inclusions (I) and nuclei. Bar = 10 µm. (B) Epi-fluorescence images of HeLa cultures infected with *C. trachomatis* L2 and cultured in the presence of DiI from 4-24 hrs post infection. Cultures were paraformaldehyde fixed at 24 hrs and counterstained with DAPI to visualize inclusions (I) and nuclei. Bar = 10 µm.


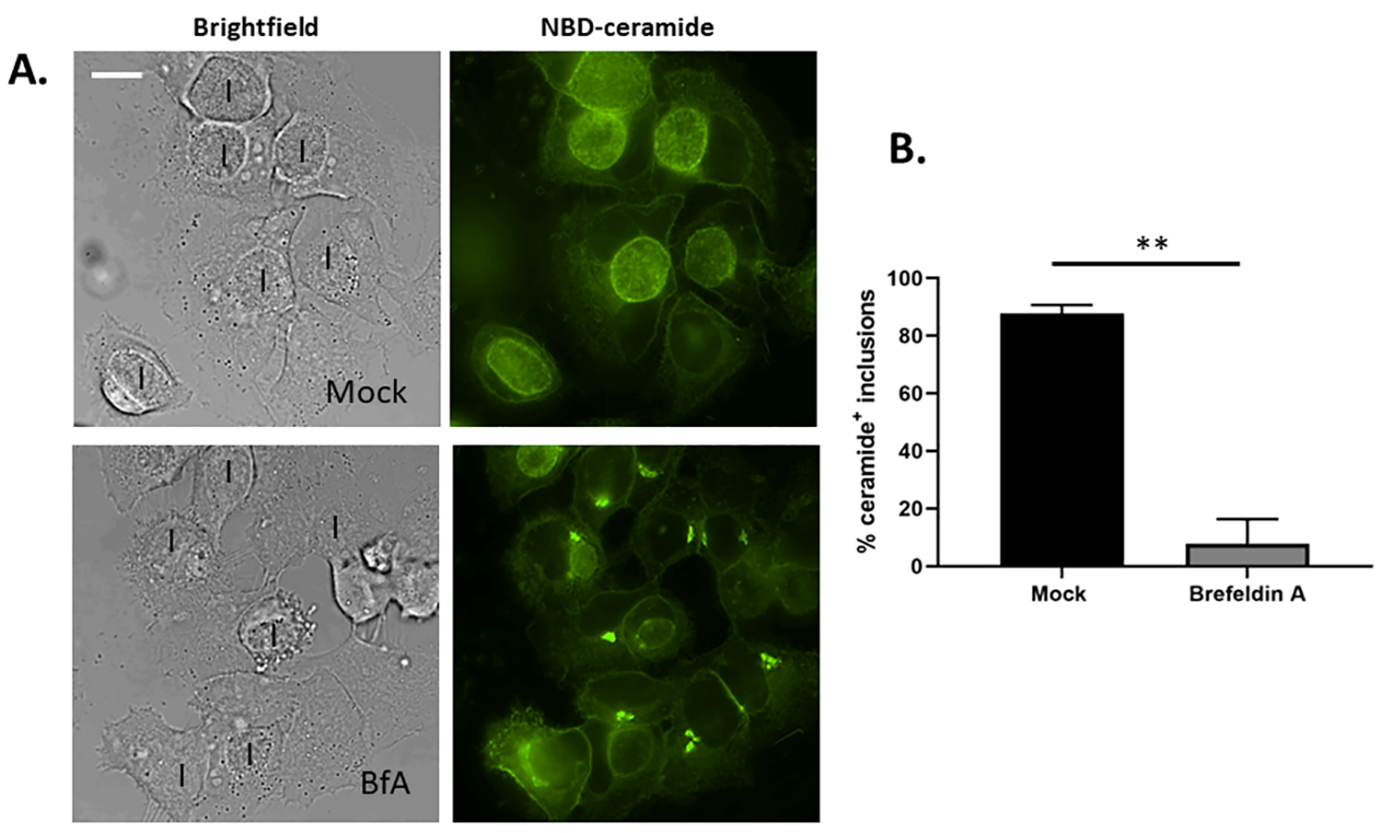


**Figure S4**. Brefeldin A interferes with NBD-ceramide labeling of inclusions. HeLa cells were infected with *C. trachomatis* L2 at an MOI of 0.5 and subsequently mock treated or treated with 3 µg/ml Brefeldin A (BfA) from 4-24hrs post infection. (A) 24 hr cultures were pulsed with ~5 µM BODIPY C_5_-Ceramide complexes and live cells were visualized after 90 min of back-extraction. Representative brightfield showing inclusions (I) and corresponding epi-fluorescence images are shown for each treatment. Bar = 10 µm. (B) Inclusions (n = 100) were manually enumerated and percent BODIPY positive inclusions are indicated. All data represented as means of triplicate samples with one standard deviation. Statistical significance was determined by Student’s T test (**, *P*<0.001).

**
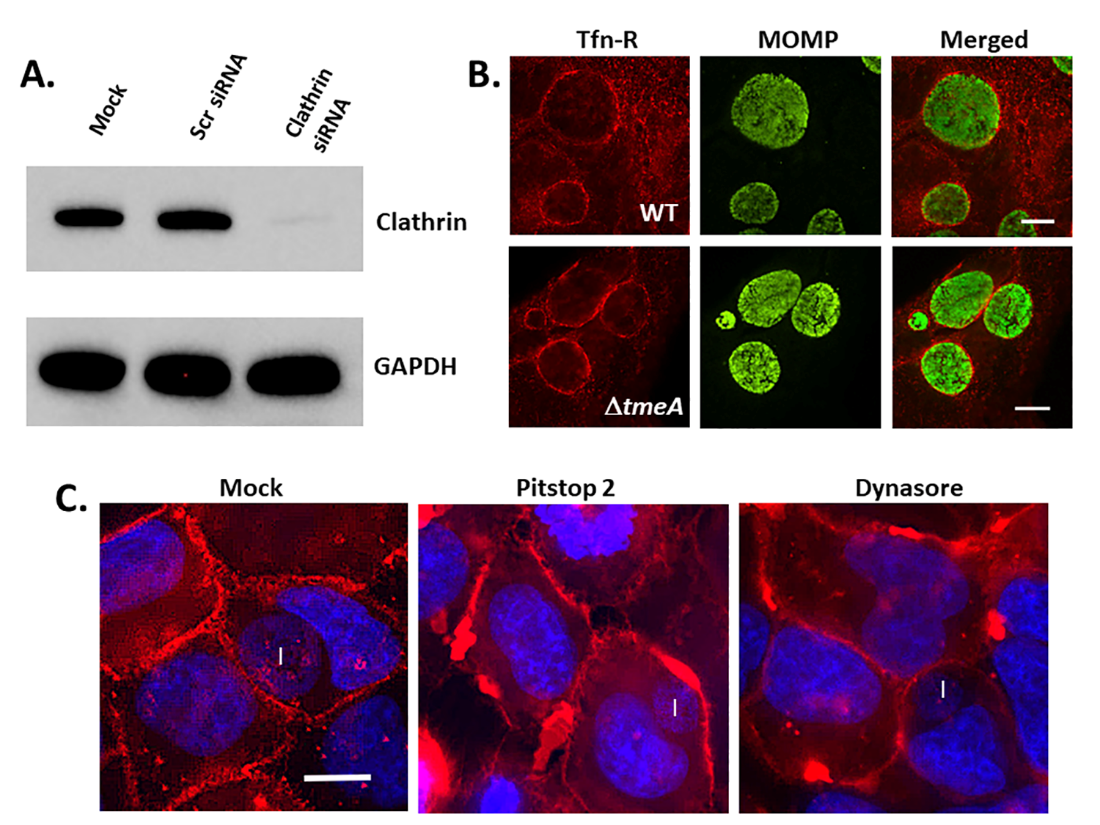
**

**Figure S5**. siRNA-mediated knockdown of clathrin and transferrin receptor localization. (A) HeLa cultures were mock treated or transfected with clathrin-specific siRNA or scramble siRNA (Scr) for 24 hr. Whole culture material was harvested and probed in immunoblots with clathrin-specific antibodies. GAPDH was probed as a loading control. (B). HeLa cells were infected at an MOI of 1 with WT or Δ*tmeA* *C. trachomatis* for 24 hrs. Cultures with methanol fixed and probed with transferrin receptor (red) or MOMP (green)-specific antibodies. (C). HeLa cells were infected with WT L2 and pulsed with DiI at 24 hrs post infection in the presence of Pitstop2 or dynasore. Cultures were immediately paraformaldehyde fixed and counter stained with DAPI to stain inclusions (I) and nuclei. Epifluorescence images are shown. Bar = 10 µm.


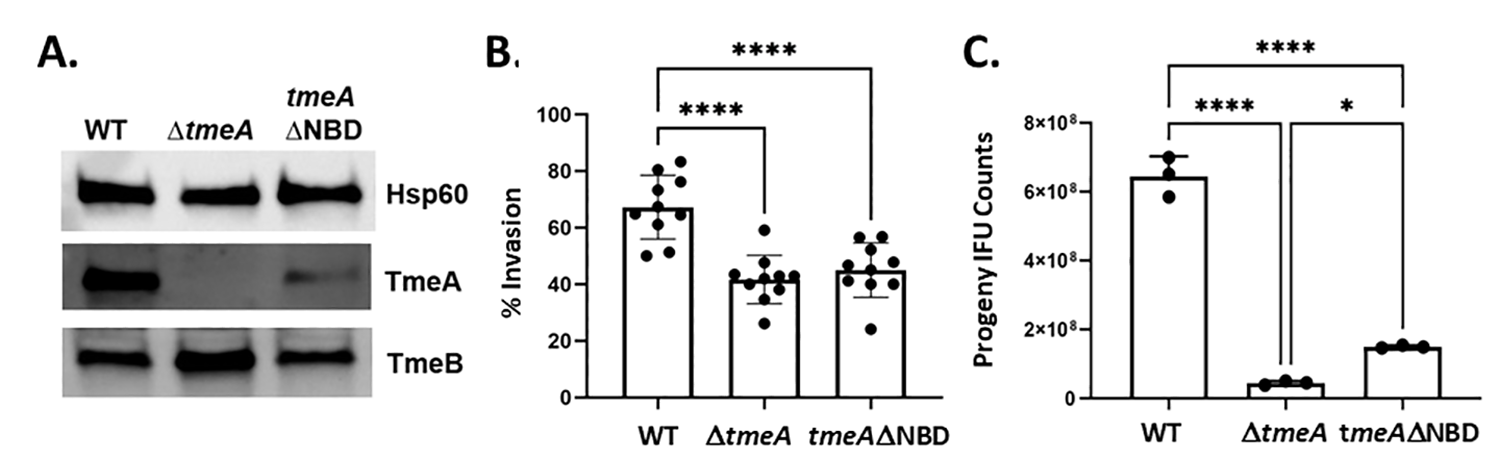


**Figure S6**. (A) Whole-culture material from HeLa equally infected for 24 hr with WT, Δ*tmeA* or *tmeA*-ΔNBD was probed in immunoblots with TmeA or TmeB-specific antibodies. Hsp60 was detected as a loading control. (B) Quantification of internalized EBs after synchronous infection at 4°C followed by shift to 37°C for 30 min. Invasion efficiencies are shown from a representative experiment as means of internalized EBs enumerated from 10 individual fields of view (closed circles). Statistical significance was determined by One-way ANOVA with multiple comparisons (****, *P*<0.0001). (C). Enumeration of progeny EBs from 24 hr HeLa cultures equivalently infected with WT, Δ*tmeA*, or *tmeA*-ΔNBD chlamydiae. Data are represented as means and standard deviation calculated from triplicate samples (closed circles). Statistical significance was determined by One-way ANOVA with multiple comparisons (*. *P*<0.01; ****, *P*<0.0001).


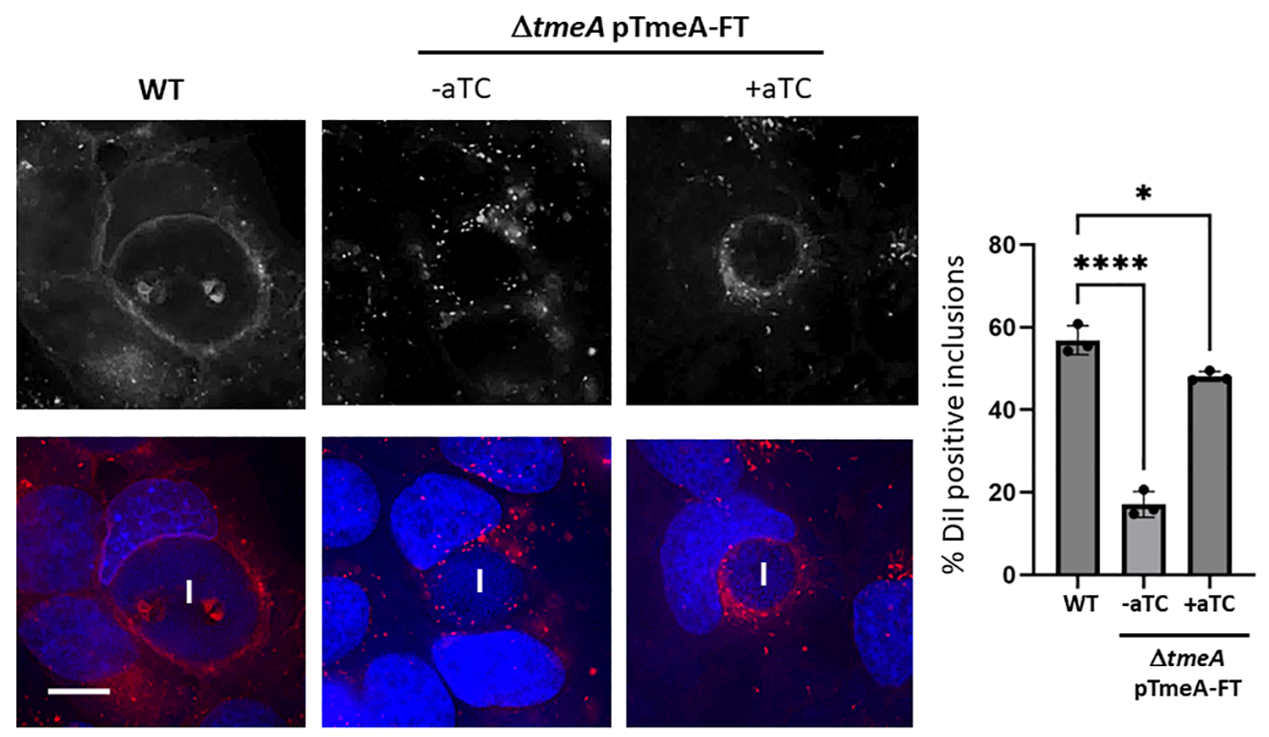


**Figure S7**. Expression of pFlag-TmeA restores DiI trafficking to Δ*tmeA* inclusions. HeLa cells were infected at an MOI of 0.1 with WT or Δ*tmeA* *C. trachomatis* expressing pFlag-TmeA. pFlag-TmeA cultures were mock treated or supplemented with 50 ng/ml aTC 5 hrs post infection. Cultures were pulsed with DiI for 90 min 24 hrs post infection. Paraformaldehyde-fixed cultures were DAPI stained (blue) and inclusions are indicated (I). Representative epifluorescence images are shown. Bar = 10 µm. DiI co-localization was assessed (n = 100) and percentages of DiI positive inclusions are represented as means of triplicate samples (closed circles) with standard deviation. Statistical significance was determined by One-way ANOVA with multiple comparisons (*. *P*<0.015; ****, *P*<0.0001).


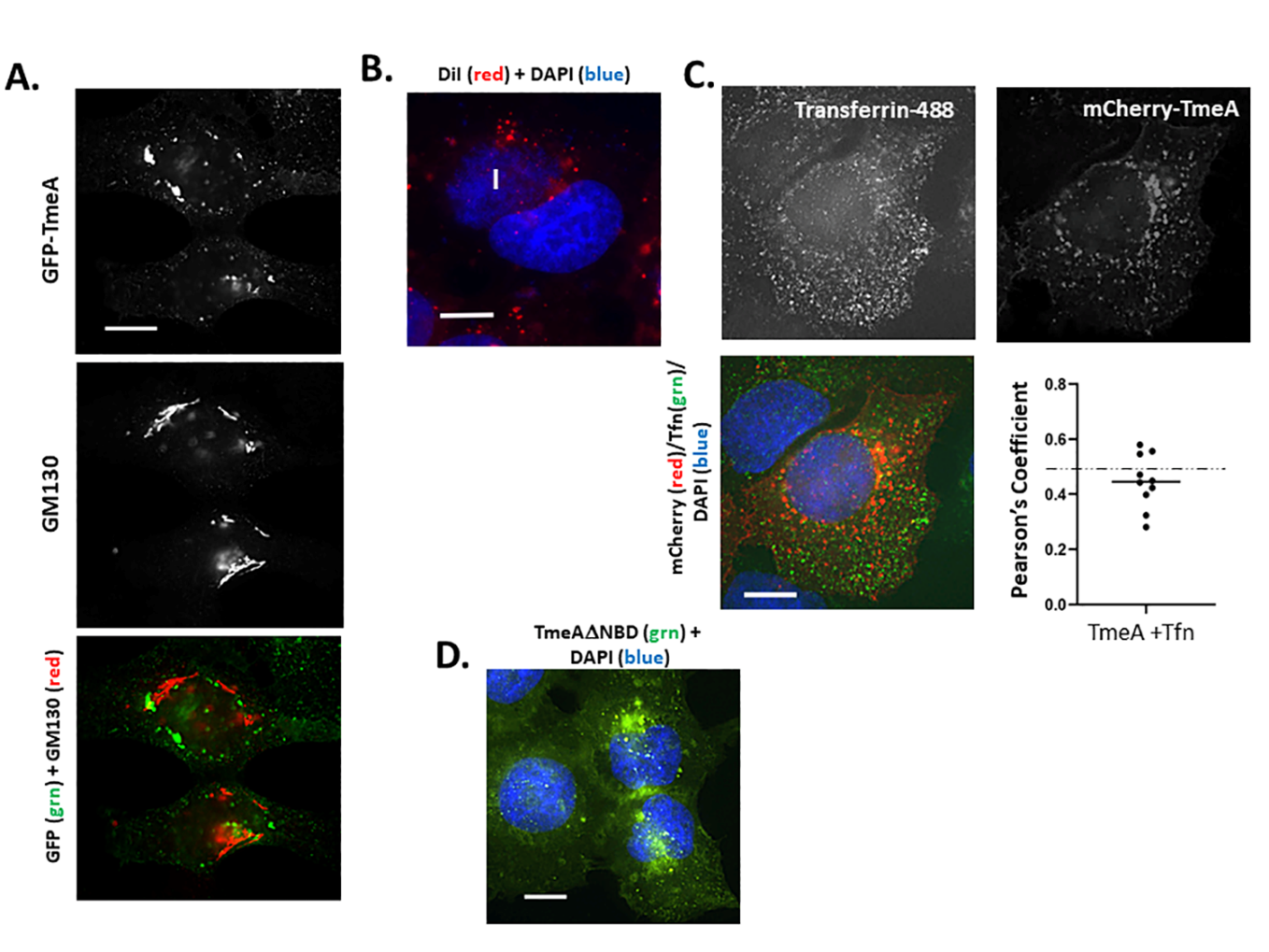


**Figure S8**. (A) Epi-fluorescence image of HeLa cells expressing GFP-TmeA (green) for 24 hrs. Cultures were fixed and Golgi structures (red) were detected by probing with GM130-specific antibodies. Single-channel and merged images are shown. Bar = 10 µm. (B) HeLa cells were infected with *C. caviae* for 24 hrs, pulsed with DiI, and paraformaldehyde fixed after 60 min. Representative epi-fluorescence images are shown for DiI (red) and DAPI (blue) with the inclusion (I) indicated in the merged image. Bar = 10 µm. (C). HeLa cells expressing mCherry-TmeA (red), for 24 hr were pulsed with Transferrin Alexa-488 (green) for 4 hrs followed by paraformaldehyde fixation. Proteins visualized by direct fluorescence and representative images are shown. Corresponding Pearson coefficient for signal overlap. Horizontal bar designates a Pearson value of 0.5. Bar = 10 µm. (D) HeLa cells were transfected with GFP-TmeAΔNBD then paraformaldehyde fixed and counterstained with DAPI at 24 hrs. A representative merged epi-fluorescence image is shown for GFP-TmeAΔNBD (green) and DAPI (blue). Bar = 10 µm.
